# ﻿Description of two new species of *Ophiocordyceps*: *O.sinocampes* and *O.cystidiata* (Ophiocordycipitaceae, Hypocreales) from typical karst landform forests in Guizhou, China

**DOI:** 10.3897/mycokeys.114.134323

**Published:** 2025-02-13

**Authors:** Zhong-Shun Xu, Li-ping Deng, Hai-Yan Wang, Hui-Ling Tian, Jiao-Jiao Qu, Yong-dong Dai, Xiao Zou

**Affiliations:** 1 Institute of Fungus Resources, Department of Ecology/Key laboratory of Plant Resource Conservation and Germplasm Innovation in Mountainous Region (Ministry of Education), College of Life Sciences, Guizhou University, Guiyang, Guizhou 550025, China Guizhou University Guiyang China; 2 School of Pharmacy, Guizhou University of Traditional Chinese Medicine, Guiyang, Guizhou, 550025, China Guizhou University of Traditional Chinese Medicine Guiyang China; 3 College of Tea Sciences, Guizhou University, Guiyang, China Guizhou University Guiyang China; 4 Fungus Medicine Research Center/ School of Basic Medical Science, Guizhou University of Traditional Chinese Medicine, Guiyang, Guizhou, 550025, China Guizhou University of Traditional Chinese Medicine Guiyang China

**Keywords:** Cordycipitoid fungi, invalidation, karst landform, nomenclature, phylogeny

## Abstract

Karst habitats are hotspots of diversity and endemism. Their naturally fragmented distributions across broad geographic landscapes have led to a complex array of smaller evolutionary ecosystems. Comprehensive biodiversity assessments of karst habitats have revealed that these ecosystems contain a high level of endemism. During a survey of cordycipitoid fungi in the karst region of southwest Guizhou, China, we identified and proposed two new species, based on morphology and multi-locus (nrSSU, nrLSU, *tef*, *rpb1*, and *rpb2*) phylogenetic analyses. *O.cystidiata***sp. nov.** is characterized by gray-white to yellow fertile part, verrucose phialides, and conidia enveloped in a cystic thickened mucus sheath, distinguishing it from other species. *O.sinocampes***sp. nov.** is distinguished by long tapering phialides with inflated bases. Phylogenetic analyses using five loci reveal that *O.cystidiata* shares a close relationship with *O.fenggangensis*, *O.musicaudata*, *O.alboperitheciata*, and *Hirsutellakuankuoshuiensis*, while *O.sinocampes* is closely related to *O.multiperitheciata* and *H.strigosa*. Herein, we completed the descriptions, illustrations and molecular phylogeny of these two new species. The host diversity of *O.sinocampes* has also been documented within the orders Lepidoptera and Coccoidea. Our research further enriches the diversity of cordycipitoid species in the typical karst landform areas of Guizhou, China.

## ﻿Introduction

Karst landform encompasses surface and subterranean features that are shaped by the action of water on soluble rocks. Karst topography represents distinctive geological formations characterized by caves, sinkholes, and other notable features. These environments harbor diverse landforms and microclimates that foster rich biological diversity. Karst habitats are hotspots of diversity and endemism due to their distinct ecological niches, which allow for the diversification of a wide variety of species (Clements et al. 2006; Grismer et al. 2021). Numerous research expeditions of karst landforms have uncovered new species with localized distributions (Tian and Huang 2015; Huang et al. 2019; Agung et al. 2021; Qu et al. 2021), identifying karsts as hotspots of endemism and biodiversity and priorities for conservation.

Guizhou, referred to as the “Karst Province of China” and “the Karst Museum”, has an extensive karst area of 128,000 km^2^, accounting for 73% of its total land area. This makes it the largest karst region in China, with diverse landscapes including peak forests, stone forests, canyons, valleys, enigmatic caves, and vast sinkholes (Li 2011). The karst landscapes of Guizhou exhibit remarkable diversity, featuring peak forests, stone forests, unique canyons, blind valleys, enigmatic caves, and vast sinkholes.

The genus *Ophiocordyceps* was established by Petch (1931) to classify the species with non-disarticulating ascospores and clavate asci with thickened apices. Most species in *Ophiocordyceps* possess firm, darkly pigmented stromata or subiculum, especially those with *Hirsutella* Pat. asexual morphs. Conversely, some species exhibit brightly or palely colored stromata associated with *Hymenostilbe* Petch and *Paraisaria* Samson & Brady asexual morphs (Mongkolsamrit et al. 2019). The stromata are generally tough, wiry, fibrous, or pliant. The perithecia, which can be superficial or completely immersed, are typically arranged obliquely or in an ordinal fashion. Ascospores in this genus are typically cylindrical and multiseptated, disarticulating into part-spores or remaining whole upon discharge (Sung et al. 2007; Xiao et al. 2023). Species of *Ophiocordyceps* are distributed worldwide across various forest ecosystems, including tropical and subtropical regions (Petch 1937; Kobayasi 1941; Tzean et al. 1997; Chen et al. 2013; Ban et al. 2014; Sanjuan et al. 2015; Luangsa-ard et al. 2018; Araújo et al. 2018; Mongkolsamrit et al. 2019; Zha et al. 2021). Notable species with widespread distribution include *O.nutans* (Friedrich et al. 2018). Among the most prominent species in this genus is *O.sinensis*, a well-known traditional Chinese medicine found exclusively in alpine environments of the Qinghai-Tibet Plateau (Dai et al. 2020). Another notable group within the genus, *O.unilateraliss.l.*, is famous for its ability to manipulate ants, turning them into “zombie ants” that facilitate the spread of spores (Evans et al. 2011). Recently, a newly discovered species, *O.megala*, was identified, notable for being the largest cordycipitoid fungus with a single specimen weighing 28 g, a hundred times heavier than typical *O.sinensis* (Dai et al. 2024).

In this study, we undertook a comprehensive survey of cordycipitoid fungi within the Xingyi karst landform area, spanning the Baishi Mountains, Malinghe Valley, and Wanfeng Forests. A total of 55 specimens were collected. One species infecting lepidopteran larvae in the Baishi Mountains was identified as *O.cystidiata*. Another specimen, parasitizing *Coccoidea* sp. from the Malinghe Valley, was validated and reclassified it as *O.sinocampes*. Morphological, microstructural, and multi-gene phylogenetic analyses were conducted to compare the new species with closely related taxa for classification and descriptions.

## ﻿Materials and methods

### ﻿Specimen collection

Specimens were collected from three karst landform areas in Xingyi City, Guizhou Province, China: (1) Baishi Mountains, Baiwanyao Village (25°4'12"N, 99°10'12"E), (2) Malinghe Valley (25°8'24"N, 104°57'36"E), and (3) Wanfeng Forests (24°59'24"N, 104°58'12"E).

### ﻿Fungal isolation and culture

Upon collection, specimens underwent surface sterilization with 75% ethanol for 1–3 min, followed by rinsing with sterile water. The internal sclerotia were isolated and cultured on potato dextrose agar (PDA) at 20 °C under dark conditions. All isolated strains were deposited at the Institute of Fungal Resources Collection, Guizhou University (GZAC), China.

### ﻿Microscopic morphological structure observation

Specimens collected in the field were analyzed and photographed using an Olympus SZ61 stereomicroscope. Fruiting bodies were sectioned and examined under a Leica S9E stereomicroscope. Sections of the fertile head were mounted on glass slides with a drop of lactic acid and lactophenol cotton blue, covered with a cover slip, and observed and photographed under a Leica DM2500 compound microscope for detailed measurements of perithecia, asci, peridium, apical cap, ascospores, and secondary ascospores.

The slant cultures were transferred to new PDA plates and incubated at 20 °C for three weeks for colony morphological observations. Circular agar blocks approximately 5 mm in diameter were extracted from a colony and transferred to fresh PDA plates for further colony morphological observations.

For the morphological description, microscope slide cultures were prepared by placing small pieces of mycelia on 5-mm diameter PDA medium blocks, which were then overlaid with a cover slip. Micro-morphological observations and measurements, including those of hyphae, phialides, and conidia, were conducted using an Olympus CX40 microscope.

### ﻿Scanning electron microscope observations

Electron microscopy was performed as previously described by Qu et al. (2021). For scanning electron microscopy (SEM) observations, 1-cm-wide agar blocks with hyphae were excised from PDA cultures. Samples were fixed with 4% glutaraldehyde overnight at 4 °C, washed thrice with phosphate buffer solution (PBS) (137 mM NaCl, 2.7 mM KCl, 8.1 mM Na_2_HPO_4_, and 1.5 mM KH_2_PO_4_; pH 7.4), each time for 10 min. Next, the fixed hyphae and conidia underwent dehydration using a series of alcohol concentrations (50%, 70%, 90%, and 100% alcohol), each step lasting 10 min.

Subsequently, the samples were dehydrated with supercritical carbon dioxide. The gold coating was then applied to the samples prior to examination. Conidia and mucilage were visualized and photographed using a Hitachi S-3400N scanning electron microscope (Japan).

### ﻿DNA extraction, polymerase chain reaction amplification, and sequencing

Genomic DNA from both the fungus and its host was extracted using a Fungi DNA isolation Kit following the manufacturer’s instructions (Transgen Bio-Tek, USA). DNA was extracted from the stroma and the surface of sclerotium sections, respectively. Additionally, genomic DNA was extracted from fungal pure cultures using 0.05–0.1 g of axenic mycelia. The concentration of the obtained genomic DNA was larger than 20 ng/μL, and this DNA was used as a template for polymerase chain reaction (PCR) amplification of target DNA fragments.

Six nuclear loci of the fungus were targeted for amplification and sequencing, namely the internal transcribed spacer (ITS), the small and large subunit ribosomal RNA (nrSSU and nrLSU, respectively), the transcription elongation factor-1 alpha (*tef*), and the largest and second largest subunits of RNA polymerase ІІ (*rpb1* and *rpb2*, respectively). The PCR assays followed protocols described by Qu et al. (2021) and Peng et al. (2024). Detailed information regarding the primers used was provided in Suppl. material 1. PCR products were subsequently sequenced using an ABI3700 automatic sequence analyzer (Sangong, Shanghai).

### ﻿Phylogenetic analysis

For the construction of a phylogenetic tree encompassing the potential new *Ophiocordyceps* species, representative taxa were selected based on five loci: nrSSU, nrLSU, *tef*, *rpb1*, and *rpb2*. These taxa were selected from previous studies within the genus *Ophiocordyceps* (Sung et al. 2007; Ban et al. 2014; Quandt et al. 2014; Sanjuan et al. 2015; Simmons et al. 2015; Luangsa-ard et al. 2018; Mongkolsamrit et al. 2019; Fan et al. 2021; Qu et al. 2021; Peng et al. 2024) (Table 1). Sequences for each locus were retrieved from GenBank using their respective accession numbers. We combined the published data with our newly generated sequences from the present study to establish a five-locus dataset. This dataset comprised a total of 212 taxa, encompassing sequence data from nrSSU, nrLSU, *tef*, *rpb1*, and *rpb2*, aimed at capturing the diversity within *Ophiocordyceps* (Table 1). As outgroups, *Drechmeriaconiospora* and *Haptocilliumsinense* were selected based on Kepler et al. (2014).

**Table 1.** Specimens and GenBank accession numbers for nrSSU, nrLSU, *tef*, *rpb1*, and *rpb2* sequences included in phylogenetic analyses.

Species	Voucher	Host	nrSSU	nrLSU	*tef1-a*	*rpb1*	*rpb2*	References
*D.balanoides*	CBS 250.82^T^	Lepidoptera	AF339539	AF339588	DQ522342	–	DQ522442	Sung et al. 2007
*D.coniospora*	ARSEF 6962	–	collected from its genome scaffold sequences (LAYC00000000)					Zhang et al. 2016
*D.gunnii*	OSC 76404	Lepidoptera	AF339572	AF339522	AY489616	AY489650	DQ522426	Kepler et al. 2012
*D.sinense*	CBS 567.95	–	AF339594	AF339545	DQ522343	DQ522389	DQ522443	Sung et al. 2001
*H.fusiformis*	ARSEF 5474	Coleoptera	KM652067	KM652110	KM651993	KM652033	–	Simmons et al. 2015
*H.gigantea*	ARSEF 30	Hymenoptera	–	JX566977	JX566980	KM652034	–	Simmons et al. 2015
*H.guyana*	ARSEF 878	Hemiptera:Cicadellidae	KM652068	KM652111	KM651994	KM652035	–	Simmons et al. 2015
*H.illustris*	ARSEF 5539	Hemiptera	KM652069	KM652112	KM651996	KM652037	–	Simmons et al. 2015
*H.kirchneri*	ARSEF 5551	Acari:Eriophyidae	KM652070	KM652113	KM651997	–	–	Simmons et al. 2015
*H.kuankuoshuiensis*	GZUIFR-2012KKS3-1	Lepidoptera larvae	–	KY415582	KY415590	KY945360	–	Qu et al. 2021
*H.lecaniicola*	ARSEF 8888	Hemiptera:Coccidae	KM652071	KM652114	KM651998	KM652038	–	Simmons et al. 2015
*H.liboensis*	ARSEF 9603	Lepidoptera: Cossidae	KM652072	KM652115	–	–	–	Simmons et al. 2015
*H.minnesotensis*	3608	*Heteroderaglycines*	collected from its genome scaffold sequences(JPUM00000000)					Lai et al. 2014
*H.nodulosa*	ARSEF 5473	Lepidoptera:Pyralidae	KM652074	KM652117	KM652000	KM652040	–	Simmons et al. 2015
*H.radiata*	ARSEF 1369	Diptera	KM652076	KM652119	KM652002	KM652042	–	Simmons et al. 2015
*H.repens* nom. *inval.*	ARSEF 2348	Hemiptera:Delphacidae	KM652077	KM652120	KM652003	–	–	Simmons et al. 2015
*H.rhossiliensis*	ARSEF 3751	–	KM652081	KM652124	KM652007	KM652046	–	Simmons et al. 2015
*H.rhossiliensis*	ARSEF 2931	Tylenchida:Heteroderidae	KM652078	KM652121	KM652004	KM652043	–	Simmons et al. 2015
*H.rhossiliensis*	ARSEF 3207	–	KM652079	KM652122	KM652005	KM652044	–	Simmons et al. 2015
*H.rhossiliensis*	ARSEF 3747	Tylenchida:Criconematidae	KM652080	KM652123	KM652006	KM652045	–	Simmons et al. 2015
*H.satumaensis*	ARSEF 996	Lepidoptera:Pyralidae	KM652082	KM652125	KM652008	KM652047	–	Simmons et al. 2015
*H.subulata*	ARSEF 2227	Lepidoptera	KM652086	KM652130	KM652013	KM652051	–	Simmons et al. 2015
*H.thompsonii*	ARSEF 256	–	KM652090	KM652135	KM652018	KM652053	–	Simmons et al. 2015
*H.thompsonii*	MTCC 3556	–	collected from its genome scaffold sequences (APKB01000000)					
*H.thompsonii*	MTCC 6686	–	collected from its genome scaffold sequence (APKU01000000)					
*H.versicolor*	ARSEF 1037	Hemiptera:Membracidae	KM652102	KM652150	KM652029	KM652063	–	Simmons et al. 2015
*H.vnecatrix*	ARSEF 5549	Ixodida	KM652073	KM652116	KM651999	KM652039	–	Simmons et al. 2015
*H.cryptosclerotium*	ARSEF 4517	Hemiptera	KM652066	KM652109	KM651992	KM652032	–	Simmons et al. 2015
*H.strigosa*	ARSEF 2197	Hemiptera: Cicadellidae	KM652085	KM652129	KM652012	KM652050	–	Simmons et al. 2015
*Hirsutella* sp.	NHJ 12525	–	–	EF469078	EF469063	EF469092	EF469111	Sung et al. 2007
*O.acicularis*	OSC 110987	Coleoptera	EF468950	EF468805	EF468744	EF468852	–	Sung et al. 2007
*O.acicularis*	OSC 110988	Coleoptera	EF468951	EF468804	EF468745	EF468853	–	Sung et al. 2007
*O.agriota*	ARSEF 5692	Coleoptera	DQ522540	DQ518754	DQ522322	DQ522368	DQ522418	Kepler et al. 2012
*O.alboperitheciata*	YHH 16755^T^	Lepidoptera	–	MT222278	MT222279	MT222280	MT222281	Fan et al. 2021
*O.aphodii*	ARSEF 5498	Coleoptera	DQ522541	DQ518755	DQ522323	–	DQ522419	Spatafora et al. 2007
*O.appendiculata*	NBRC 106959	Coleoptera	JN941729	JN941412	AB968578	JN992463	AB968540	Ban et al. 2015
*O.appendiculata*	NBRC 106960	Coleoptera	JN941728	JN941413	AB968577	JN992462	AB968539	Ban et al. 2015
*O.araracuarensis*	HUA 186148	–	KC610790	KF658679	KC610739	KF658667	KC610717	Dai et al. 2024
*O.arborescens*	NBRC 105890	Cossidae; Lepidoptera	–	AB968415	AB968573	–	AB968535	Ban et al. 2015
*O.arborescens*	NBRC 105891	Cossidae; Lepidoptera	–	AB968414	AB968572	–	AB968534	Ban et al. 2015
*O.australis*	1348a	Hymenoptera	collected from its genome scaffold sequences(NJEU00000000)					De Bekker et al. 2017
*O.australis*	Map64	Hymenoptera	collected from its genome scaffold sequences(DAJKKO000000000)					De Bekker et al. 2017
*O.bispora*	ERS1123077	Hymenoptera	collected from its genome scaffold sequences(DAJKKO000000000)					Conlon et al. 2017
*O.blakebarnesii*	MISSOU1	–	KX713644	–	KX713686	KX713713	–	Araújo et al. 2018
*O.blakebarnesii*	MISSOU3	–	KX713643	KX713608	KX713687	KX713714	–	Araújo et al. 2018
*O.brunneanigra*	BCC69032	–	–	MF614654	MF614638	MF614668	MF614681	Luangsa-ard et al. 2018
*O.brunneiperitheciata*	BCC64201	–	–	MF614658	MF614643	–	MF614685	Luangsa-ard et al. 2018
*O.brunneiperitheciata*	BCC 49312	Lepidoptera	–	MF614660	MF614642	–	MF614686	Luangsa-ard et al. 2018
*O.brunneipunctata*	OSC 128576	Coleoptera (Elateridae)	DQ522542	DQ518756	DQ522324	DQ522369	DQ522420	Spatafora et al. 2007
*O.camponoti-balzani*	G104	*Camponotusbalzani*	KX713660	KX713593	KX713703	KX713703	–	Araújo et al. 2018
*O.camponotibispinosi*	OBIS4	–	KX713637	–	KX713692	KX713720	–	Araújo et al. 2018
*O.camponoti-femorati*	FEMO2	–	KX713663	KX713590	KX713678	KX713702	–	Araújo et al. 2018
*O.camponoti-hippocrepidis*	HIPPOC	Hemiptera	KX713655	KX713597	KX713673	KX713707	–	Araújo et al. 2018
*O.camponotileonardi*	BCC 80369	–	collected from its genome scaffold sequences(PDHP01000000)					Kobmoo et al. 2019
*O.camponoti-nidulantis*	NIDUL2	–	KX713640	KX713611	KX713669	KX713717	–	Araújo et al. 2018
*O.camponoti-rufipedis*	G108	–	KX713659	KX713594	KX713679	KX713704	–	Araújo et al. 2018
*O.camponoti-rufipedis*	Map16	–	collected from its genome scaffold sequence(NJES00000000)					De Bekker et al. 2017
*O.camponoti-saundersi*	BCC 79314	–	collected from its genome scaffold sequences(PDHQ00000000)					Kobmoo et al. 2019
*O.camponoti-renggeri*	ORENG	–	KX713634	KX713617	KX713671	–	–	Araújo et al. 2018
O.cf.acicularis	OSC 128580	Coleoptera	DQ522543	DQ518757	DQ522326	DQ522371	DQ522423	Spatafora et al. 2007
*O.clavata*	NBRC 106961	–	–	JN941414	AB968586	–	AB968547	Schoch et al. 2012
*O.coccidiicola*	NBRC 100682	–	AB968391	AB968419	AB968583	–	AB968545	Ban et al. 2015
*O.cochlidiicola*	HMAS 199612	–	KJ878917	KJ878884	KJ878965	KJ878998	–	Quandt et al. 2014
*O.crinalis*	GDGM 17327	Lepidoptera	KF226253	KF226254	KF226256	KF226255		Wang et al. 2014
*O.curculionum*	OSC 151910	–	KJ878918	KJ878885		KJ878999	–	Quandt et al. 2014
***O.cystidiata***	**GZUIFR-2023XY-OA5**	**Hepialidae**	**PQ497594**	**PQ497634**	–	**PQ516632**	**PQ516636**	**This study**
***O.cystidiata***	**GZUIFR-2023XY-OA5C**	**Hepialidae**	**PQ497595**	**PQ497635**	–	**PQ516633**	**PQ516637**	**This study**
*O.desmidiospora*	SJS3Des	–	MH536515	MH536514	MN785129	MN785131	–	Saltamachia and Araújo 2020
*O.elongata*	OSC 110989	Lepidoptera	–	EF468808	EF468748	EF468856	–	Sung et al. 2007
*O.entomorrhiza*	KEW 53484	-	EF468954	EF468809	EF468749	EF468857	EF468911	Sung et al. 2007
*O.evansii*	Ophsp 858	Lepidoptera	EF468954	EF468809	EF468749	EF468857	–	Sanjuan et al. 2015
*O.fenggangensis*	FG21042850	Lepidoptera	OR527538	OR527541	OR526345	OR526350	OR526353	Peng et al. 2024
*O.fenggangensis*	HKAS 125848^T^	Lepidoptera	–	OR527542	OR526346	OR526351	–	Peng et al. 2024
*O.formicarum*	TNS F18565	–	KJ878921	KJ878888	KJ878968	KJ879002	KJ878946	Quandt et al. 2014
*O.formosana*	MFLU:15-3888	–	KU854951	–	KU854949	KU854947	–	Li et al. 2016
*O.formosana*	NTU 00035	–	–	–	KT275192	KT275190	KT275191	Wang et al. 2015a
*O.forquignonii*	OSC 151902	–	KJ878912	KJ878876	–	KJ878991	KJ878945	Quandt et al. 2014
*O.fulgoromorphila*	Ophara729	–	KC610795	KC610761	KC610730	KF658677	AB968554	Sanjuan et al. 2015
*O.geometridicola*	BCC35947	–	–	MF614647	MF614631	MF614664	MF614678	Luangsa-ard et al. 2018
*O.geometridicola*	BCC79823	–	–	MF614648	MF614632	MF614663	MF614679	Luangsa-ard et al. 2018
*O.ghanensis*	Gh41	–	KX713656	–	KX713668	KX713706	–	Araújo et al. 2018
*O.highlandensis*	HKAS83207	Scarabaeoidea	KM581284	–	–	KM581274	KM581278	Yang et al. 2015
*O.highlandensis*	YHH OH1301	Melolonthidae	KR479869	–	KR479870	KR479872	KR479874	Wang et al. 2015b
*O.irangiensis*	OSC 128578	Hymenoptera:ant	DQ522556	DQ518770	DQ522345	DQ522391	DQ522445	Spatafora et al. 2007
*O.karstii*	MFLU: 15-3884	Hepialidae	KU854952	–	KU854945	KU854943	–	Li et al. 2016
*O.karstii*	MFLU: 15-3885	Hepialidae	KU854953	–	KU854946	KU854944	–	Li et al. 2016
*O.khonkaenensis*	BCC81463	–	MK632127	MK632102	MK632076	MK632169	MK632158	Crous et al. 2019
*O.kimflemingiae*	SC09B	–	KX713631	–	KX713698	KX713724	–	Araújo et al. 2018
*O.kniphofioides*	MF90	Hymenoptera	MK874746	MK875538	–	MK863827		Araújo et al. 2018
*O.konnoana*	EFCC 7315	Coleopteran	EF468959	–	EF468753	EF468861	EF468916	Sung et al. 2007
*O.lanpingensis*	YHOL0707	Hepialidae	KC417459	KC417461	KC417463	KC417465	–	Chen et al. 2013
*O.liangii*	HKAS 125845^T^	Lepidoptera	OR527539	OR527543	OR526347	–	–	Peng et al. 2024
*O.liangii*	LB22071253	Lepidoptera	OR527540	OR527544	OR526348	–	OR526354	Peng et al. 2024
*O.liangshanensis*	YFCC 8577	Lepidoptera(Hepialidae	MT774218	MT774225	MT774246	MT774232	MT774239	Wang et al. 2021
*O.liangshanensis*	YFCC 8578	Lepidoptera(Hepialidae)	MT774219	MT774226	MT774247	MT774233	MT774240	Wang et al. 2021
*O.lloydii*	OSC 151913	Hymenoptera (Camponotus)	KJ878924	KJ878891	KJ878970	KJ879004	–	Quandt et al. 2014
*O.longissima*	NBRC 106965	–	AB968392	AB968420	AB968584	–	AB968546	Ban et al. 2015
*O.longissima*	TNS F18448	–	KJ878925	KJ878892	KJ878971	KJ879005	–	Quandt et al. 2014
*O.macroacicularis*	BCC 22918	Lepidopter	–	MF614655	MF614639	MF614669	MF614675	Luangsa-ard et al. 2018
*O.macroacicularis*	NBRC 100685	–	AB968388	AB968416	AB968574	–	AB968536	Ban et al. 2015
*O.macroacicularis*	NBRC 105888	Hepialidae	AB968389	AB968417	AB968575	–	AB968537	Ban et al. 2015
*O.macroacicularis*	NBRC 105889	Hepialidae	AB968390	AB968418	AB968576	–	AB968538	Ban et al. 2015
*O.macroacicularis*	TNS F18550	–	KJ878911	KJ878875	KJ878959	–	–	Quandt et al. 2014
*O.megala*	YHH OMYP 1507001	Hepialidae	NMDCN00011VK	NMDCN00011VM	NMDCN00011VO	NMDCN00011VQ	NMDCN00011VS	Dai et al. 2024
*O.megala*	YFCC OMLP15079192	Hepialidae	NMDCN00011VL	NMDCN00011VN	NMDCN00011VP	NMDCN00011VR	NMDCN00011VT	Dai et al. 2024
*O.melolonthae*	Ophgrc679	–	–	KC610768	KC610744	KF658666	–	Sanjuan et al. 2015
*O.melolonthae*	OSC 110993	Coleoptera	–	–	DQ522331	DQ522376	–	Spatafora et al. 2007
*O.monacidis*	MF74	Hymenoptera	KX713647	KX713605	–	KX713712	–	Araújo et al. 2018
*O.multiperitheciata*	BCC 22861	Lepidoptera	–	MF614656	MF614640	MF614670	MF614683	Luangsa-ard et al. 2018
*O.multiperitheciata*	BCC 69008	Lepidoptera	–	MF614657	MF614641	–	MF614682	Luangsa-ard et al. 2018
*O.musicaudata*	SY22072879	Lepidoptera	–	OR527545	OR526349	OR526352	–	Peng et al. 2024
*O.myrmecophila*	CEM1710	–	KJ878928	KJ878894	KJ878974	KJ879008	–	Peng et al. 2024
*O.myrmecophila*	TNS 27120	–	KJ878929	KJ878895	KJ878975	KJ879009	–	Quandt et al. 2014
*O.naomipierceae*	DAWKSANT	Hymenoptera	KX713664	KX713589	–	KX713701	–	Araújo et al. 2018
*O.neovolkiana*	OSC 151903	–	KJ878930	KJ878896	KJ878976	–	–	Quandt et al. 2014
*O. nigre1la*	EFCC 9247	Lepidoptera	EF468963	EF468818	EF468758	EF468866	EF468920	Sung et al. 2007
*O.nooreniae*	BRIP 55363	Hymenoptera	KX673811	KX673810	KX673812	–	KX673809	Crous et al. 2016
*O.nujiangensis*	YFCC8880	Hepialidae	ON723384	ON723381	ON868820	ON868823	ON868826	Sun et al. 2022
*O.nujiangensis*	YHH 20041	Lepidoptera	ON723385	ON723383	ON868822	ON868825	ON868827	Sun et al. 2022
*O.nutans*	NBRC 100944	–	JN941713	JN941428	AB968588	–	AB968549	Ban et al. 2015
*O.nutans*	OSC 110994	stink bug	DQ522549	DQ518763	DQ522333	DQ522378	–	Spatafora et al. 2007
*O.ootakii*	J13	Hymenoptera (Polyrhachis moesta)	KX713652	KX713600	KX713681	KX713708	–	Araújo et al. 2018
*O.ovatospora*	YHH2206001	–	–	OP295113	OP313801	OP313803	OP313805	Tang et al. 2022
*O.ovatospora*	YFCC22069184	–	OP295111	OP295114	OP313802	OP313804	–	Tang et al. 2022
*O.pauciovoperitheciata*	BCC39781	–	–	MF614650	MF614635	MF614667	MF614671	Luangsa-ard et al. 2018
*O.pauciovoperitheciata*	BCC45562	–	–	MF614651	MF614634	MF614666	MF614674	Luangsa-ard et al. 2018
*O.polyrhachis-furcata*	BCC 54312	–	collected from its genome scaffold sequences(LKCN00000000)					Wichadakul et al. 2015
*O.ponerus*	XCH ant 03	Hymenoptera	KY953152	–	KY953153	KY953154	–	Qu et al. 2018
*O.pruinosa*	NHJ 12994	Hemiptera	EU369106	EU369041	EU369024	EU369063	EU369084	Johnson et al. 2009
*O.pseudoacicularis*	BCC49256	Hymenoptera: ant	–	MF614645	MF614629	MF614662	MF614676	Luangsa-ard et al. 2018
*O.pseudoacicularis*	BCC53843	Hymenoptera: ant	–	MF614646	MF614630	MF614661	MF614677	Luangsa-ard et al. 2018
*O.pulvinata*	TNS F 30044	Hymenoptera: ant	GU904208	AB721305	GU904209	GU904210	–	Kepler et al. 2011
*O.purpureostromata*	TNS F18430	Coleoptera	KJ878931	KJ878897	KJ878977	KJ879011	–	Quandt et al. 2014
*O.ramosissimum*	GZUH2012HN2	*Endoclita* sp. (Hepialidae)	KJ028013	–	KJ028016	KJ028018	–	Wen et al. 2014
*O.ramosissimum*	GZUHHN8	*Phassusnodus*	KJ028012	–	KJ028014	KJ028017	–	Wen et al. 2014
*O.ravenelii*	OSC 151914	–	KJ878932	–	KJ878978	KJ879012	KJ878950	Quandt et al. 2014
*O.robertsii*	UoM1	Hepialidae	collected from its genome scaffold sequences(JAPEBV000000000)					Xu et al. 2023
*O.robertsii*	UoM4	Hepialidae	collected from its genome scaffold sequences(JAPEBW000000000)					Xu et al. 2023
*O.rubiginosiperitheciata*	NBRC 100946	–	JN941705	JN941436	AB968581	JN992439	AB968543	Schoch et al. 2012
*O.rubiginosiperitheciata*	NBRC 106966	–	JN941704	JN941437	AB968582	JN992438	AB968544	Schoch et al. 2012
*O.salganeicola*	JPMA107	–	MT741703	MT741716	MT759574	MT759577	–	Araujo et al. 2020
*O.salganeicola*	Mori01	–	MT741705	MT741719	MT759575	MT759578	MT759580	Araujo et al. 2020
*O.satoi*	J19	*Polyrhachislamellidens*	KX713650	KX713601	KX713684	KX713710	–	Araújo et al. 2018
*O.sinensis*	QH06-197	Hepialidae	JX968025	JX968030	JX968015	JX968005	JX968010	Zhang et al. 2013
*O.sinensis*	QH09-201	Hepialidae	JX968024	JX968029	JX968014	JX968004	JX968009	Zhang et al. 2013
*O.sinensis*	XZ06-44	Hepialidae	JX968026	JX968031	JX968016	JX968006	JX968011	Zhang et al. 2013
*O.sinensis*	YN07-8	Hepialidae	JX968027	JX968032	JX968017	JX968007	JX968012	Zhang et al. 2013
*O.sinensis*	YN09-64	Hepialidae	JX968028	JX968033	JX968018	JX968008	JX968013	Zhang et al. 2013
*O.sinensis*	CO18	Hepialidae	collected from its genome scaffold sequences(ANOV00000000)					
*O.sinensis*	CUHK CSC2	Hepialidae	–	HM595902	HM595936	HM595968	–	Chan et al. 2011
*O.sinensis*	ZJB12195	Hepialidae	collected from its genome scaffold sequences(LWBQ01000000)					
***O.sinocampes***	**GZUIFR 2010MC-1**	**Lepidoptera**	–	**PQ766190**	**PQ787212**	–	**PQ787213**	**This study**
***O.sinocampes***	**GZUIFR-2022MLH-H1**	**Coccoidea**	**PQ497592**	**PQ497632**	**PQ516628**	**PQ516630**	**PQ516634**	**This study**
***O.sinocampes***	**GZUIFR-2022MLH-H1C**	**Coccoidea**	**PQ497593**	**PQ497633**	**PQ516629**	**PQ516631**	**PQ516635**	**This study**
*O.sobolifera*	KEW 78842	Cicadidae	EF468972	EF468828	–	EF468875	EF468925	Sung et al. 2007
*O.sobolifera*	NBRC 106967	Cicadidae	AB968395	AB968422	AB968590	–	AB968551	Ban et al. 2015
*O.spataforae*	MY11765	–	–	MG831747	MG831746	MG831748	MG831749	Luangsa-ard et al. 2018
*O.spataforae*	OSC 128575	Hemiptera	EF469126	EF469079	EF469064	EF469093	EF469110	Sung et al. 2007
*O.sphecocephala*	NBRC 101416	–	JN941698	JN941443		JN992432	–	Schoch et al. 2012
*O.spicatus* sp. nov.	MFLU18-0164	Coleoptera: Tenebrionoidea	MK863047	MK863054	MK860192	–	–	Zha et al. 2021
*O.stylophora*	OSC 111000	Coleoptera (Elateridae	DQ522552	DQ518766	DQ522337	DQ522382	DQ522433	Spatafora et al. 2007
*O.stylophora*	NBRC 100947	–	JN941694	JN941447	AB968579	JN992428	AB968541	Schoch et al. 2012
*O.stylophora*	NBRC 100948	–	JN941693	JN941448	AB968580	JN992427	AB968542	Schoch et al. 2012
*O.stylophora*	NBRC 100949	–	JN941692	JN941449	–	JN992426	–	Schoch et al. 2012
*O.stylophora*	OSC 110999	–	EF468982	EF468837	EF468777	EF468882	EF468931	Sung et al. 2007
*O.stylophora*	OSC111000	Coleoptera	DQ522552	DQ518766	DQ522337	DQ522382	DQ522433	Spatafora et al. 2007
*O.thanathonensis*	MFU16-2909	–	–	MF850378	MF872613	MF872615	–	Xiao et al. 2017
*O.tiputini*	Ophsp. 11465	–	KC610792	KC610773	KC610745	KF658671	–	Sanjuan et al. 2015
*O.tricentri*	NBRC 106968	–	AB968393	AB968423	AB968593	–	AB968554	Ban et al. 2015
*O.unilateralis*	Ophuni866	–	KC610799	–	KC610742	KF658674	KC610718	Sanjuan et al. 2015
*O.unilateralis*	OSC 128574	Hymenoptera	DQ522554	DQ518768	DQ522339	DQ522385	DQ522436	Spatafora et al. 2007
*O.unilateralis*	SC16a	–	collected from its genome scaffold sequences(LAZP02000001)					De Bekker et al. 2015
*O.unilateralis*	SERI1	*Camponotussericeiventris*	KX713628	KX713626	KX713675	KX713730	–	Araújo et al. 2018
*O.unitubercula*	YFCC HU1301	Lepidoptera: Noctuidae	KY923213	KY923211	KY923215	KY923217	–	Wang et al. 2018
*O.unitubercula*	YHH HU1301	Lepidoptera: Noctuidae	KY923214	KY923212	KY923216	KY923218	–	Wang et al. 2018
*O.variabilis*	ARSEF 5365	Dipteran	DQ522555	DQ518769	DQ522340	DQ522386	DQ522437	Spatafora et al. 2007
*O.xuefengensis*	GZUH2012HN13	*Phassusnodus*	KC631787	–	KC631792	KC631797	–	Wen et al. 2013
*O.xuefengensis*	GZUH2012HN14^T^	*Phassusnodus*	KC631789	–	KC631793	KC631798	–	Wen et al. 2013
*O.yakusimensis*	HMAS _199604	Cicadidae	KJ878938	KJ878902	–	KJ879018	KJ878953	Quandt et al. 2014
*Ophiocordyceps* sp1.	HKAS125843	–	–	OQ110570	OQ116920	OQ116923	–	unpublished
*Ophiocordyceps* sp1.	HKAS125849	–	–	OQ110571	OQ116921	OQ116924	–	unpublished
*Ophiocordyceps* sp1.	HKAS125850	–	–	OQ110572	OQ116922	OQ116925	–	unpublished
*Ophiocordyceps* sp2.	TNS 16250	Coleoptera	KJ878942	–	KJ878987	KJ879021	–	Quandt et al. 2014
*Ophiocordyceps* sp2.	TNS 16252	–	KJ878941	KJ878906	KJ878986	–	–	Quandt et al. 2014
*Ophiocordyceps* sp3.	NHJ 12581	Lepidoptera	EF468973	EF468831	EF468775	–	EF468930	Sung et al. 2007
*Ophiocordyceps* sp3.	NHJ 12582	Lepidoptera	EF468975	EF468830	EF468771	–	EF468926	Sung et al. 2007
*Ophiocordyceps* sp4.	OSC 110997	–	EF468976	–	EF468774	EF468879	EF468929	Sung et al. 2007
*par. amazonica*	Ophama2026	–	KJ917562	KJ917571	KM411989	KP212902	KM411982	Sanjuan et al. 2015
*par. blattarioides*	HUA 186093	Blattodea	KJ917559	KJ917570	KM411992	KP212910	–	Sanjuan et al. 2015
*Par. Coenomyiae*	NBRC 108993	–	AB968384	AB968412	AB968570	–	AB968532	Ban et al. 2015
*par. gracilioides*	Ophgrc934	–	KJ917556	–	–	KP212914	KP212914	Sanjuan et al. 2015
*par. gracilis*	EFCC 3101	Lepidoptera	EF468955	EF468810	EF468750	EF468858	EF468913	Sung et al. 2007
*par. gracilis*	EFCC 8572	Lepidoptera	EF468956	EF468811	EF468751	EF468859	EF468912	Sung et al. 2007
*par. heteropoda*	NBRC 100642	–	JN941720	JN941421	AB968594	–	AB968555	Ban et al. 2015
*par. orthopterorum*	BBC88305	Orthoptera (nymph)	–	MK332583	MK214080	MK214084	–	Mongkolsamrit et al. 2019
*par. phuwiangensis*	TBRC9709	Coleoptera; Elateridae	–	MK192057	MK214082	MK214086	–	Mongkolsamrit et al. 2019
*par. tettigonia*	GZUHCS14062709	Tettigoniidae sp.	KT345955	–	KT375440	KT375441	–	Wen et al. 2014
*par. yodhathaii*	BBH43163	Coleoptera; Elateridae	–	MK332584	MH211353	MH211353	–	Mongkolsamrit et al. 2019
*Podonectriacitrina*	TNSF18537	–	–	KJ878903	KJ878983	–	KJ878954	Quandt et al. 2014
*Stilbellabuquetii*	HMAS199617	–	KJ878940	KJ878905	KJ878985	KJ879020	–	Quandt et al. 2014

The alignment of nrSSU and nrLSU sequences was performed using MAFFT (Katoh et al. 2002) with default settings. For the exon regions of *tef*, *rpb1*, and *rpb2*, alignment was conducted using codon models. The total alignment lengths for the five loci were as follows: 1060 bp for nrSSU; 968 bp for nrLSU; 936 bp for *tef*; 555 bp for *rpb1* and 936 bp for *rpb2*, resulting in a combined dataset length of 4455 bp. All five loci were integrated into a unified dataset, which was further partitioned into 11 distinct segments for analysis. This partitioning included one segment each for nrSSU and nrLSU, along with nine additional segments corresponding to the three codon positions within the protein-coding genes *tef*, *rpb1*, and *rpb2*.

The optimal partitioning scheme and evolutionary models for the 11 predefined partitions were determined using PartitionFinder2 (Lanfear et al. 2016), employing a greedy algorithm and the Akaike information criterion. The analysis yielded the following 10 partitions with their respective best-fit models: Partition 1—nrSSU: TRNEF+I+G, Partition 2—nrLSU, Partitions 3–5—*tef* codon1, codon 2 and codon 3: GTR+I+G, Partition 6—*rpb1* codon1, *rpb2* codon1: TVM+I+G; Partitions 7–9 —*rpb1* codon2, codon3 and *rpb2* codon2: GTR+I+G, and Partition 10— *rpb2* codon3: TIM+I+G.

The maximum likelihood phylogenetic tree was constructed using IQ-TREE (Nguyen et al. 2015) with 1000 ultrafast bootstrap replicates (Minh et al. 2013). The Shimodaira–Hasegawa-like approximate likelihood ratio test was employed to assess branch support (Guindon et al. 2010). The entire phylogenetic analysis was performed using PhyloSuite v1.2.2 (Zhang et al. 2020).

The Bayesian inference phylogenetic tree was constructed using MrBayes 3.2.6 (Ronquist et al. 2012) with a partition model. The analysis involved running two parallel Markov Chain Monte Carlo (MCMC) runs for 50,000,000 generations. The substitution model settings (lset) used the general time reversible model (nst = 6) and a gamma distribution of rate variation across sites (rates = invgamma), which was applied uniformly across all 10 partitions. To ensure the robustness of the phylogenetic inference, the initial 25% of sampled data were discarded as burn-in. The convergence of MCMC chains was monitored throughout the analysis, and the operation was stopped when the average standard deviation of split frequencies fell below 0.01, indicating convergence. Due to the extensive dataset and the time-consuming process, we employed the CIPRES Science Gateway (https://www.phylo.org/portal2/) to conduct the Bayesian phylogenetic analysis. The consensus tree was visualized and analyzed for tree topology and branch support using FigTree v.1.6 (http://tree.bio.ed.ac.uk/software/figtree/). While ITS sequences were not used to build the phylogenetic tree, they helped distinguish the relationships between the two novel taxa and closely related species.

## ﻿Results

### ﻿Phylogenetic analysis

**Figure 1.**
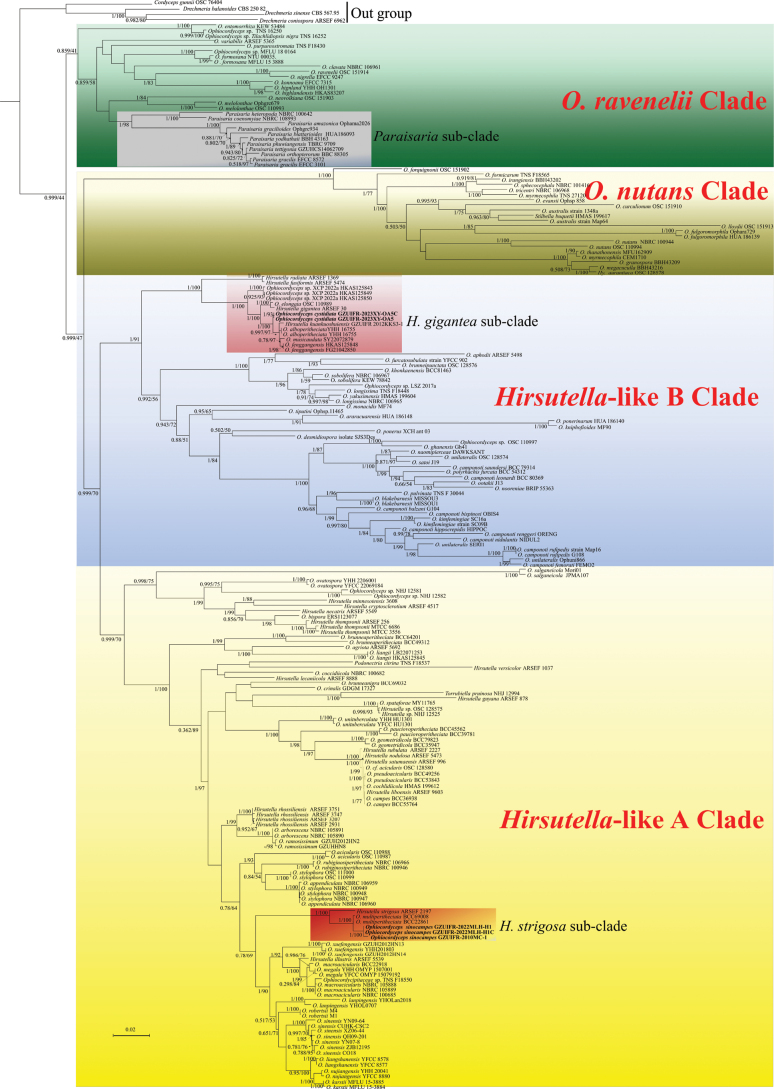
The phylogeny of *Ophiocordyceps* with emphasis on *O.sinocampes*, *O.cystidiata* and their related species based on 5-locus (nrSSU, nrLSU, *tef*, *rpb1*, and *rpb2*) datasets.

A total of 213 taxa were classified into five well-supported clades within *Ophiocordyceps* based on the combined five-locus dataset (nrSSU, nrLSU, *tef*, *rpb1*, and *rpb2*) using maximum likelihood (ML) and Bayesian inference (BI) analyses. These clades were designated here as the *Hirsutella*-like A clade (BI = 0.999), *Hirsutella*-like B clade (BI = 1.00), *O.nutans* clade (BI = 1.00), and *O.ravenelii* clade (BI = 0.859) (Fig. 1). *O.sinocampes* was found to belong to the *Hirsutella*-like A clade, clustering within the *H.strigosa* sub-clade. It was identified as a sister species to *O.multiperitheciata*. The separate clade with high support values highlighted the distinctiveness of *O.sinocampes* from its closely related species. Similarly, *O.cystidiata* was positioned within the *Hirsutella*-like B clade, specifically clustering into the *H.gigantea* sub-clade. It was found to be a sister species to *O.fenggangensis*, *O.musicaudata*, *O.alboperitheciata*, and *H.kuankuoshuiensis*. The distinct clade formed by *O.cystidiata* with high support values underscores its differentiation from other species within the sub-clade.

### ﻿Taxonomy

#### ﻿ Ophiocordyceps sinocampes

Taxon classificationFungiHypocrealesOphiocordycipitaceae

X. Zou, Zhong S. Xu & J.J. Qu sp. nov.

54C66E13-7FC2-5C73-8D79-1A53465B864D

 854597

Fig. 2

##### Synonym.

*Hirsutellacampes* nom. invalid. X. Zou, J.J. Qu, Y.F. Han & Z.Q. Liang, Journal of Mountain Agriculture and Biology 40(6): 1–12, 2021 (in Chinese).

##### Etymology.

The name *sinocampes* was derived from “sino,” referring to China, and “campes”, referring to the host in Latin, meaning caterpillar.

##### Holotype.

**Figure 2.**
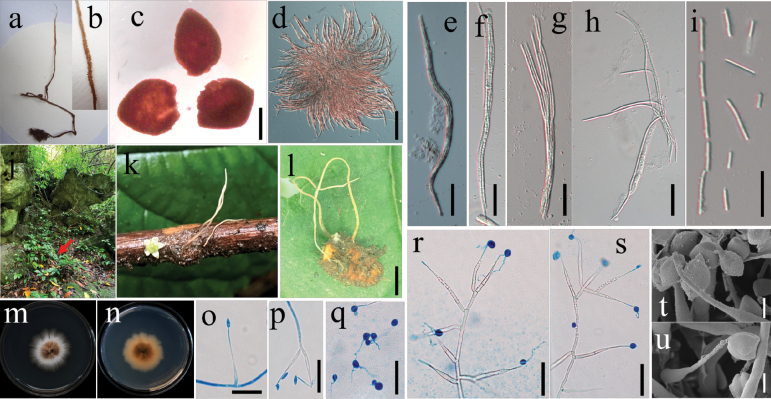
Morphological characteristics of *O.sinocampes***a–i** morphological and micromorphological characteristics of specimen GZUIFR-2010MC **c** perithecia **d–i** ascus and ascospore **j–t** morphological characteristics of specimen GZUIFR-2022MLH-H1 and its pure culture GZUIFR-2022MLH-H1C **j–l** wild morph **m, n** fungus in culture **o–t** phialides and conidia. Scale bars: 150 μm (**c**); 50 μm (**d**); 30 μm (**e–i**); 15 μm (**o–s**); 5 μm (**t, u**).

GZUIFR-2010MC(Fig. 2a), China • Guizhou Province: Kuankuoshui National Nature Reserve (28°6'36"N, 107°2'24"E). The specimen was found on the larva of Lepidoptera buried in soil, collected in July 2010 by X. Zou (ex-holotype: GZUIFR-2010MC-1) (The GenBank accession number of ITS: PQ765882; nrLSU: PQ766190; tef: PQ787212; rpb2: PQ787213).

##### Host.

The larvae of Lepidoptera.

##### Description.

***Stromata***: Single, clavate, solid, lignified, yellow-brown, arising from the head of the host, 120–150 × 0.5–1.0 mm. ***Fertile part***: Cylindrical, yellowish, about 5 cm long. ***Perithecia***: Superficial, ovoid, 320–350 × 260–300 μm. ***Asci***: Cylindrical, hyaline, 8-spored, 130–210 × 4–6 μm, with the apex thickened to form a hemispherical ascus cap that is, measuring 5–5.5 × 3.2–4.0 μm. ***Ascospores***: Filiform, hyaline, irregular, multi-septate, disarticulating into secondary ascospores, 4.5–11 × 1.5–2.0 µm.

***Asexual morph***: *Hirsutella*-like.

***Colonies***: The colony reaches 13–18 mm in diameter after two weeks on PDA at 22 °C, appearing round with irregular swellings. The edge of the colony is fluffy, with a slight yellow protrusion in the middle and dark brown pigment secreted on the back, measuring approximately 10–15 mm in diameter. ***Hyphae***: Hyaline, smooth-walled, septate, branched, 1.8–3.6 μm wide. ***Conidiogenous cells***: Monophialidic, hyaline, smooth-walled, subulate, growing directly or laterally from hyphae, tapering gradually into a slender neck (21.6–38.4 µm long). The base width measures 2.4–4.8 µm, and the neck width measures 0.9–1.5 µm. ***Conidia***: Hyaline, smooth, arising solitarily from the apex of conidiogenous cells, oval or orange-like shape, often enveloped in a mucous sheath, usually single, rarely aggregated in pairs or triplets (6–8.4 × 2.9–4.3 µm).

##### Distribution.

China, Guizhou Province: Zunyi and Xingyi City.

##### Additional specimens examined.

GZUIFR-2022MLH-H1 (Fig. 2l), and its pure culture GZUIFR-2022MLH-H1C, China. Guizhou Province: Malinghe Valley, Xingyi City (25°8'24"N, 104°57'36"E; altitude, 1068 m). These specimens were found on a larva of Coccoidea in soil, collected in July 2022 by Xiao Zou, Jiaojiao Qu, and Zhongshun Xu.

##### Notes.

The basionym of *O.sinocampes* is *H.campes*, which was initially documented in the Journal of Mountain Agriculture and Biology (in Chinese) in 2021(Table 2). Notably, the taxonomic validity of *H.campes* is compromised due to its description being solely in Chinese, which does not meet the requisite standards set forth by the International Code of Nomenclature for algae, fungi, and plants (ICN). According to the ICN, the descriptions of new species must be provided in English or Latin (McNeill et al. 2012).

**Table 2.** Morphological comparison of *O.sinocampes* and its relatives.

Species	Host	Habitat	Stromata (mm)	Perithecium (μm)	Asci (μm)	Ascospore (μm)	Colony (mm)	Conidiogenous cells (μm)	Conidia (μm)	References
*O.sinocampes*	Homoptera, Coccoidea	The karst-landform forest of Xingyi City, Guizhou Province, China.	Single, clavate, solid, lignified, yellow-brown, arising from the head of the host, 120–150 × 0.5–1.0	300-350 × 210-290	110-230 × 4-7	6-12 × 1.5–2.5	reaches 13–18 in diameter after two weeks on PDA at 22 °C, appearing round with irregular swellings. fluffy, with a slight yellow protrusion in the middle and dark brown pigment secreted on the back	Monophialidic, hyaline, smooth-walled, growing directly or laterally from hyphae.	Smooth, oval or orange-like shape, often enveloped in a mucous sheath, usually single, rarely aggregated in pairs or triplets (6–8.4 × 2.9–4.3)	In this study
	Lepidopteran caterpillars	Kuankuoshui National Nature Reserve	–	–	–	–	villous and yellowish in the middle. The color is convex, the back secretes dark brown pigment, and the diffusion circle is large, 10–15	The base is cylindrical or conical, 21.6 –38.4	fusiform or orange-petaled, 6–8.4 × 2.9–4.3	In this study; Zou et al. 2021a
*O.multiperitheciata*	Hepialidae	On Lepidoptera larva in the leaf litter of forest floor.	Several,cylindrical, branched dentritic, 75–110 long, 1–1.5 wide, dark brown to black	superficial, gregarious, distributing unequally on upper of the stromata, ordinal in arrangement, narrowly ovoid, brown to dark brown, 990–1200 × 350–450	Asci hyaline, cylindrical, 8-spores, 400–600 × 6–7.5	hyaline, filiform, 470–660 × 1.5–2.5, remaining whole after discharge, multiseptate	Colonies on PDA growing slowly, flat and velvety in the middle, attaining a diameter of 16–22 within 20 d at 20 °C.	monophialidic or polyphialidic,arising from hyphae laterally or terminally, hyaline, cylindrical to lanceolate,tapering gradually or abruptly into a long slender neck	hyaline, 1-celled, smooth walled, oval to lemon shaped, 8–14 × 5–8, embedded in a mucous sheath.	Luangsa-ard et al. 2018
*H.strigosa*	Cicadellidae, Homoptera	–	–	–	–	–	–	swollen in basal portion, 4.5–7.2 × 1.4–2.5, and tapering to 0.4–0.9 wide and 6.3–14.4 overall length	Conidia Cymbiform or orange segments 8.0–12.0 × 3.0–5.0	Petch 1939
*H.shennongjiaensis*	Earwig, Dermaptera	Shennongjia Forest Area	Synnemata cylindrical, size 60.0 × 1.0–2.0, brown	–	–	–	Colonies diam. up to 24.0–32.0 after 30 d at 16 °C on PDA agar, white or brown, flat, felty, the middle light‐brown part with cashmere, reverse orange yellow to light‐brown	Conidiogenous cells solitary, phialides cylindrical or awllike, 14.4–26.1 or 6.3–14.4	Conidia, hyaline,aseptate, smooth, sausage-shaped, single or double from the apex of the neck, 6.3–10.8 × 3.6–6.3	Zou et al. 2016a

Furthermore, based on the priority under ICN, the genus *Hirsutella* has been considered as a synonym of the genus *Ophiocordyceps* (Quandt et al. 2014). Through morphological and five-gene phylogenetic analyses, it is more appropriate to assign this species to *Ophiocordyceps*. Since the name *O.campes* was already used by Tasanathai et al. (2020), we renamed our species as *O.sinocampes*.

In this study, we described the sexual stage, completing the species’ sexual and asexual stage descriptions. Additionally, a new specimen of this species was reported in the karst landform area— Malinghe Valley, Xingyi, enhancing our understanding of the species’ hosts and habitats.

*O.sinocampes* is closely related to *O.multiperitheciata* Tasan., Thanakitp., Khons. & Luangsa-ard (Luangsa-ard et al. 2018) and *H.strigosa* (Petch 1939). Morphologically, *O.sinocampes* is similar to *H.strigosa* due to the long and base-inflated phialides, but it differs in having tapering phialides of *O.sinocampes*.

#### ﻿ Ophiocordyceps cystidiata

Taxon classificationFungiHypocrealesOphiocordycipitaceae

X. Zou, Zhong S. Xu & Y.D. Dai sp. nov.

CC463EB1-B4A6-5162-94A6-54DE5F44A9E2

 854598

Fig. 3

##### Etymology.

*Cystidiata* refers to the saccate mucous sheath that envelopes the conidium.

##### Holotype.

**Figure 3.**
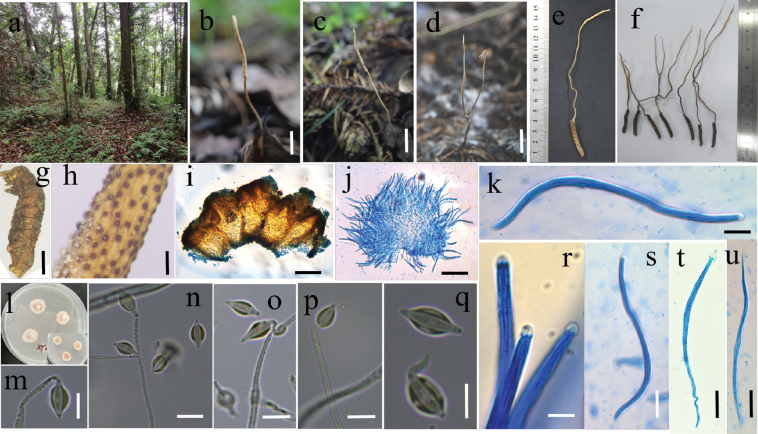
The morphological and micromorphological characteristics of *O.cystidiata***a** wild environment of *O.cystidiata***b–f** Wild morph **g** host **h, i** superficial perithecia **j, k, r–u** ascus and ascospore **i** colony **m–q** phialide and conidium. Scale bars: 1 cm (**g**); 1 mm (**h**); 100 μm (**i–j**); 20 μm (**k, r–u**); 5 μm (**m–q**).

GZUIFR-2023XY-OA5 (Fig. 3b), China • Guizhou Province: Baishi Mountains, Baiwanyao Village, Xingyi City (25°4'12"N, 99°10'12"E; altitude, 1720 m). These specimens were found on a larva of Hepialidae, buried in soil, collected in July 2023 by Zhongshun Xu, Binghui Zhou, Yongdong Dai, Huiling Tian, and Xiao Zou (ex-holotype: GZUIFR-2023XY-OA5C). (The GenBank accession numbers: nrSSU, PQ497594; nrLSU, PQ497634; rpb1, PQ516632; rpb2, PQ516636).

##### Host.

The larvae of Hepialidae (Lepidoptera).

##### Description.

***Stromata***: Arising from the head the host, lignified, solitary, rarely branched, brown to yellow-brown, 60–146 mm long. ***Fertile part***: Cylindrical, yellowish, without a sterile tip, surface spinous due to protruding ostioles, up to 18 × (0.8-) 1.44 mm.

***Perithecia***: Immersed, ovoid to oblong-ovate, 355–434 × 178–220 μm. ***Asci***: Cylindrical, hyaline, eight-spored ascus, 133–224 × 5–7 μm, apex thickened to form an ascus cap, hemispherical, 4.7–5.6 × 3.6–4.0 μm. ***Ascospores***: Filiform, hyaline, irregular, multi-septate, non-disarticulating, 13.2–25.2 × 1.5–2.6 μm, with septa.

***Asexual morph***: *Hirsutella*-like

***Colonies***: On PDA, reaching 13–16 mm in diameter after two weeks at 20 °C, round, irregularly swollen, initially light yellow, gradually changing color with an outer layer of white, an inner layer of rose red, and a central white protrusion. The back of the colony is rose red. ***Hyphae***: The basal hyphae are hyaline, smooth-walled, and septate; the apical hyphae are verrucose (2.5–3.3 μm wide). ***Conidiogenous cells***: Growing from verrucose apical hyphae, monophialidic, 22–56 µm long. The base is cylindrical, with an inflated structure near the bottom, tapering gradually into a slender neck. The base width measures 2.4–3.3 µm, and the neck width measures 0.9–1.4 µm. ***Conidia***: Arising solitarily from the apex of conidiogenous cells, fusiform or orange-like shape, usually solitary, hyaline, smooth-walled, measuring 10–12 × 2.5–3.2 µm, often enveloped in a hyaline mucous sheath (1.5–3.0 µm thick).

##### Distribution and habitat.

The karst-landform forest of Xingyi City, Guizhou Province, China.

##### Additional specimens examined.

GZUIFR-2023XY-OA2, 3, 6, 7, 8, 9, 10, 11 (Fig. 3f). Location: China. Guizhou Province: Baishi Mountains, Baiwanyao Village, Xingyi City (25°4'12"N, 99°10'12"E; altitude: 1710–1730 m). These specimens were found on a larva of *Hepialidae* sp. buried in soil, collected in July 2023 by Zhongshun Xu, Binghui Zhou, Yong-dong Dai, Huiling Tian, and Xiao Zou.

##### Notes.

*O.cystidiata* is closely related to *O.fenggangensis* (Peng et al. 2024), *O.musicaudata* (Peng et al. 2024), *O.alboperitheciata* (Fan et al. 2021), and *H.kuankuoshuiensis* (Qu et al. 2021). Morphologically, *O.cystidiata* is similar to *O.fenggangensis* and *O.musicaudata* in the shape of the stromata, fertile part, and perithecia but it differs by its inconspicuous separate ascospores (Table 3). It also resembles *H.kuankuoshuiensis* in asexual morphology but differs in having phialides with a verrucose apex and conidia enveloped by a thickened mucous sheath.

**Table 3.** Morphological comparison of *O.cystidiata* and its relatives.

Species	Host	Habitat	Stromata (mm)	Perithecium (μm)	Asci (μm)	Ascospore (μm)	Colony (mm)	Conidiogenous cells (μm)	Conidia (μm)	References
*O.cystidiata*	Hepialidae sp	Karst-landform forest	Arising from the head of host, lignified, solitary, rare branched, brown to yellow-brown, 60–146 long	Immersed, ovoid to oblong-ovate, 355–434 × 178–220	Cylindrical, hyaline, eight-spored ascus, 133–224 × 5–7, apex thickened to form ascus cap, hemispherical, 4.7–5.6 × 3.6–4.0	Filiform, hyaline, irregular, multi-septate, non-disarticulating, 13.2–25.2 × 1.5–2.6 septa	13–16 in diameter after two weeks at 20 °C, round, irregularly swollen, initially light yellow, gradually with an outer layer of white, an inner layer of rose red, and a central white protrusion. Rose red of back	Monophialidic, 22–56 long. The base is cylindrical, with a clearly inflated structure near the bottom, tapering gradually into a slender neck. The base 2.4–3.3 wide, and neck 0.9–1.4 wide.	Fusiform or orange-like shape, usually solitary, hyaline, smooth-walled, measuring10–12 × 2.5–3.2, often enveloped in a hyaline mucous sheath (1.5–3.0 thick).	In this study
*O.fenggangensis*	Lepidoptera	–	102 × 1–1.5, solitary, cylindrical, brown to off-white	306–496 × 134–223, immersed, off-white to yellowish, ovoid to oblong-ovate	91–176 × 2–8, cylindrical, apex thickened	0.3–0.7 wide, filiform, hyaline, disarticulating, secondary ascospores 2.8–6.0 × 0.3–0.7, cylindrical	–	–	–	Peng et al. 2024
*O.musicaudata*	Lasiocampidae, Lepidoptera	–	130–140 × 1–2, solitary or numerous, simple or branched, cylindrical, brown to yellowish	260–492 × 144–314, immersed, yellowish, flask-shaped	123–264 × 5–13, filiform, cylindrical, 8-spored, usually without thickened Apices	114–298 × 1.5–4.0, cylindrical, Irregular multi-septate, non disarticulating	–	–	–	Peng et al. 2024
*O.alboperitheciata*	Noctuidae, Lepidoptera	Buried in fallen leaves	Stromata in pairs, rigid, the stalk is smooth, unbranched, long 54–65, light brown to dark brown, with a clavate fertile part, white to light brown, 4.1–4.5 × 0.8–1.4, and a sterile tip.	Superficial, scattered or crowded, 410–550 230–320, nearly ovoid, white nearly light brown.	Asci hyaline, cylindrical, 8-spores, 144–246 × 3.5–4.7, with a hemispheric apical cap, 3.2–4.2 × 2.3–2.5	Hyaline, cylindric, multiseptate, 0.5–0.6 diameter, with septa 1.1–1.3 apart	–	–	–	Fan et al. 2021
*O.elongata*	Apalela americana (Lepidoptera)	–	The stalk is flexuose, longitudinally sulcate and twisted, 110 long, pale brown.	The perithecia are immersed, scattered or crowded, ovato conoid, size 50 × 30 mm, apex subacute, wall vellow by transmitted light.	The asci are 220 long, 8 diameter.	Cylindric,2 diameter, with septa 4–12 apart.	–	–	–	Fan et al. 2021
*H.kuankuoshuiensis*	Lepidoptera sp.	–	Synnemata are single, extending from the head of insect; 86 long, dark brown and changing to brown towards the apex; no conidiation was observed	–	–	–	Centre of surface with brown dense bulges and grey-white sparse flocculent aerial hyphae. Colony margin is flat with radial groove; the back of colony appears dark brown; thickness 10–12	Monophialidic, hyaline, borne perpendicular or at an acute angle to the subtending hyphae	Clavate, narrow fusiform or botuliform without a diaphragm, 9.9–12.6 × 2.7–4.5, single-or double-enveloped in a hyaline mucus, thickness 2.0–3.0	Qu et al. 2021

## ﻿Discussion

*Ophiocordycepssinocampes* was originally discovered on a caterpillar in the Kuankuoshui National Nature Reserve, a non-karst landform area in Guizhou. In this study, we present a new specimen parasitizing *Coccoidea* sp. (Hemiptera), a typical karst landform environment. Despite significant differences in host and ecological habitat, no distinguishing differences were observed in the asexual morph. Furthermore, the specimens were identical in their ITS, nrLSU, and *tef* sequences. The initial discrepancy in the nrLSU sequence (GenBank No. MF623040) was attributed to machine misreading. The only two mutations presented in the *rpb2* gene are synonymous: site 369 (TTG/TTA) [Leu] and site 564 (GTT/GTC) [Val] (Suppl. material 2). Based on the morphological indistinction and the highly consistent sequences across multiple loci, the evidence strongly supports that both specimens belong to the same species-*O.sinocampes*, underscoring the importance of molecular data in species identification.

Moreover, host jumping is also widely discovered in cordycipitiod fungi, such as *Beauveria*, *Metarhizium* and ant-infecting *Ophiocordyceps* (Pu et al. 2013; Lin et al. 2020; Patil et al. 2021). The discovery of the host transboundary of *O.sinocampes* provides further evidence of this phenomenon in *Ophiocordyceps* species. However, considering the differences in hosts, it is necessary to conduct omics analyses of the two strains in the future.

*O.cystidiata* is a recently identified species found in the Xingyi karst landform area. It is characterized by its gray-white to yellow fertile part, verrucose phialides, and conidia enveloped in a cystic mucous sheath. Among *Ophiocordyceps* species with *Hirsutella*-like characteristics and mucous sheaths outside the conidia, notable examples include *H.shennongjiaensis* (Zou et al. 2016a), *H.liboensis* (Zou et al. 2010), *H.nodulosa*, *H.tortricicola* (Zou et al. 2016b), and *H.kuankuoshuiensis* (Qu et al. 2021). However, the mucous sheath of *O.cystidiata* is thicker and darker, giving its entire structure the appearance of a typical lantern.

Karst landform areas represent a distinct geological feature and habitat type, making the investigation of cordycipitoid fungus biodiversity in these regions highly significant. Zhu et al. (2004) summarized 45 cordycipitoid species found in the karst areas of Guizhou, Yunnan, and Guangxi, highlighting the richness of resources in these landscapes. Chen et al. (2022) conducted a study in the Monkey-Ear Tiankeng karst region (Kaiyang, Guizhou Province), reporting 15 species, including 8 new *Cordyceps*-like fungi, which further illustrated the abundance of cordycipitoid fungi in the Karst Tiankeng. Our present study surveyed cordycipitoid resources in the typical karst landform areas of Xingyi, resulting in the identification of two new species. Karst ecosystems are more fragile than other ecosystems, with low stability and high vulnerability to disturbance. Consequently, conservation is crucial to safeguard the biodiversity and ecological functions of karst environments.

## Supplementary Material

XML Treatment for Ophiocordyceps sinocampes

XML Treatment for Ophiocordyceps cystidiata

## ﻿Additional information

### Author ORCIDs

Zhong-Shun Xu https://orcid.org/0009-0004-3307-4978

Li-ping Deng https://orcid.org/0009-0003-2486-7098

Hai-Yan Wang https://orcid.org/0000-0001-9190-0490

Hui-Ling Tian https://orcid.org/0009-0001-0385-9721

Jiao-Jiao Qu https://orcid.org/0000-0002-6401-4052

Yong-dong Dai https://orcid.org/0000-0001-8262-9803

Xiao Zou https://orcid.org/0000-0002-3666-5536

### Data availability

All sequences were submitted to GenBank and obtained the accession number. The alignment dataset of multigene, and the phylogenetic tree files were submitted to figshare. https://doi.org/10.6084/m9.figshare.28043432.v2.

## Supplementary materials

Supplementary material 1 Primer pairs for gene amplification and sequencing used in this study Data type xlsx https://binary.pensoft.net/file/1250054This dataset is made available under the Open Database License (http://opendatacommons.org/licenses/odbl/1.0/). The Open Database License (ODbL) is a license agreement intended to allow users to freely share, modify, and use this Dataset while maintaining this same freedom for others, provided that the original source and author(s) are credited.Zhong-Shun Xu, Li-ping Deng, Hai-Yan Wang, Hui-Ling Tian, Jiao-Jiao Qu, Yong-dong Dai, Xiao Zou

Supplementary material 2 Identification and recognition of mutation in two strains of *Ophiocordycepssinocampes* Data type png https://binary.pensoft.net/file/1250055This dataset is made available under the Open Database License (http://opendatacommons.org/licenses/odbl/1.0/). The Open Database License (ODbL) is a license agreement intended to allow users to freely share, modify, and use this Dataset while maintaining this same freedom for others, provided that the original source and author(s) are credited.Zhong-Shun Xu, Li-ping Deng, Hai-Yan Wang, Hui-Ling Tian, Jiao-Jiao Qu, Yong-dong Dai, Xiao Zou

## References

[B1] Agung AP Grismer LL Grismer JL Quah ES Chornelia A Lu JM Hughes AC (2021) A new species of *Hemiphyllodactylus* Bleeker (Squamata: Gekkonidae) from Yunnan, China and its phylogenetic relationship to other congeners. Zootaxa 4980(1): 1–27. 10.11646/zootaxa.4980.1.1 34186994

[B2] Araújo JP Evans HC Kepler R Hughes DP (2018) Zombie-ant fungi across continents: 15 new species and new combinations within *Ophiocordyceps*. I. Myrmecophilous hirsutelloidÂ species. Studies in Mycology 90(1): 119–160. 10.1016/j.simyco.2017.12.002 29910522 PMC6002356

[B3] Araujo J Moriguchi GM Uchiyama S Kinjo N Matsuura Y (2020) Insights into the Ecology and Evolution of Blattodea-associated *Ophiocordyceps*. Research Squarehttps, 1–21. 10.21203/rs.3.rs-68793/v1

[B4] Ban N Schmidli J Schär C (2014) Evaluation of the convection-resolving regional climate modeling approach in decade-long simulations. Journal of Geophysical Research. Atmospheres 119: 7889–7907. 10.1002/2014JD021478

[B5] Ban S Sakane T Nakagiri A (2015) Three new species of *Ophiocordyceps* and overview of anamorph types in the genus and the family Ophiocordycipitaceae. Mycological Progress 14: 1017–1028. 10.1007/s11557-014-1017-8

[B6] Chan WH Ling KH Chiu SW Shaw PC But PH (2011) Molecular analyses of *Cordycepsgunnii* in China. Journal of Food and drug Analysis 19(1): 6. 10.38212/2224-6614.2184

[B7] Chen ZH Dai YD Yu H Yang K Yang ZL Yuan F Zeng WB (2013) Systematic analyses of *Ophiocordycepslanpingensis* sp. nov., a new species of *Ophiocordyceps* in China. Microbiological Research 168(8): 525–532. 10.1016/j.micres.2013.02.010 23578962

[B8] Chen WH Liang JD Ren XX Zhao JH Han YF Liang ZQ (2022) Species Diversity of *Cordyceps*-Like Fungi in the Tiankeng Karst Region of China. Microbiology Spectrum 10(5): e0197522. 10.1128/spectrum.01975-22 PMC9603550 36094103

[B9] Clements R Sodhi NS Schilthuizen M Ng PK (2006) Limestone karsts of Southeast Asia: Imperiled arks of biodiversity. Bioscience 56(9): 733–742. 10.1641/0006-3568(2006)56[733:LKOSAI]2.0.CO;2

[B10] Conlon BH Mitchell J De Beer ZW Carøe C Gilbert MTP Eilenberg J Poulsen M de Fine Licht HH (2017) Draft genome of the fungus-growing termite pathogenic fungus *Ophiocordycepsbispora* (Ophiocordycipitaceae, Hypocreales, Ascomycota). Data in Brief 11: 537–542. 10.1016/j.dib.2017.02.051 28349099 PMC5357700

[B11] Crous PW Wingfield MJ Burgess TI Hardy GSJ Crane C Barrett S Cano-Lira JF Le Roux JJ Thangavel R Guarro J Stchigel AM Martín MP Alfredo DS Barber PA Barreto PW Baseia IG Cano-Canals J Cheewangkoon R Ferreira RJ Gené J Lechat C Moreno G Roets F Shivas RG Sousa JO Tan YP Wiederhold NP Abell SE Accioly T Albizu JL Alves JL Antoniolli ZI Aplin N Araújo J Arzanlou M Bezerra JDP Bouchara JP Carlavilla JR Castillo A Castroagudín VL Ceresini PC Claridge GF Coelho G Coimbra VRM Costa LA da Cunha KC da Silva SS Daniel R de Beer ZW Dueñas M Edwards J Enwistle P Fiuza PO Fournier J García D Gibertoni TB Giraud S Guevara-Suarez M Gusmão LFP Haituk S Heykoop M Hirooka Y Hofmann TA Houbraken J Hughes DP Kautmanová I Koppel O Koukol O Larsson E Latha KPD Lee DH Lisboa DO Lisboa WS López-Villalba Á Maciel JLN Manimohan P Manjón JL Marincowitz S Marney TS Meijer M Miller AN Olariaga I Paiva LM Piepenbring M Poveda-Molero JC Raj KNA Raja HA Rougeron A Salcedo I Samadi R Santos TAB Scarlett K Seifert KA Shuttleworth LA Silva GA Silva M Siqueira JPZ Souza-Motta CM Stephenson SL Sutton DA Tamakeaw N Telleria MT Valenzuela-Lopez N Viljoen A Visagie CM Vizzini A Wartchow F Wingfield BD Yurchenko E Zamora JC Groenewald JZ (2016) Fungal Planet description sheets: 469–557. Persoonia. Persoonia 37(1): 218–403. 10.3767/003158516X694499 28232766 PMC5315290

[B12] Crous PW Wingfield MJ Lombard L Roets F Swart WJ Alvarado P Carnegie AJ Moreno G Luangsaard J Thangavel R Alexandrova AV Baseia IG Bellanger JM Bessette AE Bessette AR De la Peña-Lastra S García D Gené J Pham THG Heykoop M Malysheva E Malysheva V Martín MP Morozova OV Noisripoom W Overton BE Rea AE Sewall BJ Smith ME Smyth CW Tasanathai K Visagie CM Adamčík S Alves A Andrade JP Aninat MJ Araújo RVB Bordallo JJ Boufleur T Baroncelli R Barreto RW Bolin J Cabero J Caboň M Cafà G Caffot MLH Cai L Carlavilla JR Chávez R de Castro RRL Delgat L Deschuyteneer D Dios MM Domínguez LS Evans HC Eyssartier G Ferreira BW Figueiredo CN Liu F Fournier J Galli-Terasawa LV Gil-Durán C Glienke C Gonçalves MFM Gryta H Guarro J Himaman W Hywel-Jones N Iturrieta-González I Ivanushkina NE Jargeat P Khalid AN Khan J Kiran M Kiss L Kochkina GA Kolařík M Kubátová A Lodge DJ Loizides M Luque D Manjón JL Marbach PAS Massola Jr NS Mata M Miller AN Mongkolsamrit S Moreau PA Morte A Mujic A Navarro-Ródenas A Németh MZ Nóbrega TF Nováková A Olariaga I Ozerskaya SM Palma MA Petters-Vandresen DAL Piontelli E Popov ES Rodríguez A Requejo Ó Rodrigues ACM Rong IH Roux J Seifert KA Silva BDB Sklenář F Smith JA Sousa JO Souza HG De Souza JT Švec K Tanchaud P Tanney JB Terasawa F Thanakitpipattana D Torres-Garcia D Vaca I Vaghefi N van Iperen AL Vasilenko OV Verbeken A Yilmaz N Zamora JC Zapata M Jurjević Ž Groenewald JZ (2019) Fungal Planet description sheets: 951–1041. Persoonia 43: 223–425. 10.3767/persoonia.2019.43.06 32214501 PMC7085856

[B13] Dai YD Wu CK Yuan F Wang YB Huang LD Chen ZH Zeng WB Wang Y Yang ZL Zeng PS Lemetti P Mo XX Yu H (2020) Evolutionary biogeography on *Ophiocordycepssinensis*: An indicator of molecular phylogeny to geochronological and ecological exchanges. Geoscience Frontiers 11(3): 807–820. 10.1016/j.gsf.2019.09.001

[B14] Dai YD Chen SQ Wang YB Wang Y Yang ZL Yu H (2024) Molecular phylogenetics of the *Ophiocordycepssinensis*-species complex lineage (Ascomycota, Hypocreales), with the discovery of new species and predictions of species distribution. IMA Fungus 15: 2. 10.1186/s43008-023-00131-8 PMC10858606 38336758

[B15] De Bekker C Ohm RA Loreto RG Sebastian A Albert I Merrow M Hughes DP (2015) Gene expression during zombie ant biting behavior reflects the complexity underlying fungal parasitic behavioral manipulation. BMC Genomics 16: 1–23. 10.1186/s12864-015-1812-x 26285697 PMC4545319

[B16] Evans HC Elliot SL Hughes DP (2011) *Ophiocordycepsunilateralis*: A keystone species for unraveling ecosystem functioning and biodiversity of fungi in tropical forests? Communicative & Integrative Biology 4(5): 598–602. 10.4161/cib.4.5.16721 PMC3204140 22046474

[B17] Fan Q Wang YB Zhang GD Tang DX Yu H (2021) Multigene phylogeny and morphology of *Ophiocordycepsalboperitheciata* sp. nov., a new entomopathogenic fungus attacking Lepidopteran larva from Yunnan, China. Mycobiology 49(2): 133–141. 10.1080/12298093.2021.1903130 37970184 PMC10635235

[B18] Friedrich RCS Shrestha B Salvador-Montoya C Tomé LM Reck M Goes-Neto A Drechsler-Santos E (2018) *Ophiocordycepsneonutans* sp. nov. a new neotropical species from *O.nutans* complex (Ophiocordycipitaceae, Ascomycota). Phytotaxa 344(3): 215–227. 10.11646/phytotaxa.344.3.2

[B19] Grismer L Wood Jr PL Poyarkov NA Le MD Karunarathna S Chomdej S Grismer JL (2021) Karstic landscapes are foci of species diversity in the world’s third-largest vertebrate genus *Cyrtodactylus* Gray, 1827 (Reptilia: Squamata; Gekkonidae). Diversity 13(5): 183. 10.3390/d13050183

[B20] Guindon S Dufayard JF Lefort V Anisimova M Hordijk W Gascuel O (2010) New algorithms and methods to estimate maximum-likelihood phylogenies: Assessing the performance of PhyML 3.0. Systematic Biology 59(3): 307–321. 10.1093/sysbio/syq010 20525638

[B21] Huang TF Zhang PL Huang XL Wu T Gong XY Zhang YX Peng QZ Liu ZX (2019) A new cave-dwelling blind loach, *Triplophysaerythraea* sp. nov. (Cypriniformes: Nemacheilidae), from Hunan Province, China. Zoological Research 40(4): 331. 10.24272/j.issn.2095-8137.2019.049 31310067 PMC6680126

[B22] Johnson D Sung GH Hywel-Jones NL Luangsa-ard JJ Bischoff JF Kepler RM Spatafora JW (2009) Systematics and evolution of the genus *Torrubiella* (Hypocreales, Ascomycota). Mycological Research 113(3): 279–289. 10.1016/j.mycres.2008.09.008 18938242

[B23] Katoh K Misawa K Kuma K Miyata T (2002) MAFFT: A novel method for rapid multiple sequence alignment based on fast Fourier transform. Nucleic Acids Research 30: 3059–3066. 10.1093/nar/gkf436 12136088 PMC135756

[B24] Kepler RM Kaitsu Y Tanaka E Shimano S Spatafora JW (2011) *Ophiocordycepspulvinata* sp. nov., a pathogen with a reduced stroma. Mycoscience 52(1): 39–47. 10.1007/S10267-010-0072-5

[B25] Kepler R Sung GH Ban S Nakagiri A Chen MJ Huang B Li ZZ Spatafora JW (2012) New teleomorph combinations in the entomopathogenic genus *Metacordyceps*. Mycologia 104(1): 182–197. 10.3852/11-070 22067304

[B26] Kepler RM Humber RA Bischoff JF Rehner SA (2014) Clarification of generic and species boundaries for *Metarhizium* and related fungi through phylogenetics. Mycologia 106(4): 811–829. 10.3852/13-319 24891418

[B27] Khonsanit A Luangsa-Ard JJ Thanakitpipattana D Kobmoo N Piasai O (2019) Cryptic species within Ophiocordycepsmyrmecophila complex on formicine ants from Thailand. Mycological Progress 18: 147–161. 10.1007/s11557-018-1412-7

[B28] Kobayasi Y (1941) The genus *Cordyceps* and its allies. Science Reports of the Tokyo Bunrika Daigaku. Section B 84: 53–260.

[B29] Kobmoo N Mongkolsamrit S Arnamnart N Luangsa-ard JJ Giraud T (2019) Population genomics revealed cryptic species within host-specific zombie-ant fungi (*Ophiocordycepsunilateralis*). Molecular Phylogenetics and Evolution 140: 106580. 10.1016/j.ympev.2019.106580 31419479

[B30] Lai Y Liu K Zhang X Zhang X Li K Wang N Shu C Wu Y Wang C Bushley KE Xiang M Liu X (2014) Comparative genomics and transcriptomics analyses reveal divergent lifestyle features of nematode endoparasitic fungus *Hirsutellaminnesotensis*. Genome Biology and Evolution 6(11): 3077–3093. 10.1093/gbe/evu241 PMC4255773 25359922

[B31] Lanfear R Frandsen PB Wright AM Senfeld T Calcott B (2016) PartitionFinder 2: New methods for selecting partitioned models of evolution formolecular and morphological phylogenetic analyses. Molecular Biology and Evolution 34(3): 772–773. 10.1093/molbev/msw260 28013191

[B32] Li ZF (2011) Division of Karst Landform in Guizhou. Guizhou Geology 28(3): 6.

[B33] Li GJ Hyde KD Zhao RL Hongsanan S Abdel-Aziz F Abdel-Wahab M Alvarado P Alves-Silva G Ammirati J Ariyawansa H Baghela A Bahkali A Beug MW Bhat DJ Bojantchev D Boonpratuang T Bulgakov T Erio C Boro MC Ceska O Chakraborty D Chen JJ Kandawatte TC Chomnunti P Consiglio G Cui BK Dai DQ Dai YC Daranagama DA Das K Dayarathne M Crop ED Oliveira R Fragoso de Souza CA Ivanildo de Souza J Dentinger BTM Dissanayake AJ Doilom M Drechsler-Santos ER Ghobad-Nejhad M Gilmore SP Góes-Neto A Gorczak M Haitjema CH Hapuarachchi K Hashimoto A He MQ Henske JK Hirayama K Iribarren MJ Jayasiri S Jayawardena RS Jeon SJ Jerônimo GH Lucia de Jesus A Jones EBG Kang JC Karunarathna SC Kirk PM Konta S Kuhnert E Langer EJ Lee HS Lee HB Li WJ Li XH Liimatainen K Lima D Lin CG Liu JK Liu X Liu ZY Luangsa-ard JJ Lücking R Lumbsch T Lumyong S Leano E Marano AV Matsumura M Mckenzie E Mongkolsamrit S Mortimer PE Nguyen TTT Niskanen T Norphanphoun C O’Malley MA Parnmen S Pawłowska J Perera RH Phookamsak R Phukhamsakda C Zottarelli C Raspé O Reck MA Rocha SCO Santiago A Senanayake I Setti L Shang QJ Singh S Sir EB Solomon KV Song J Srikitikulchai P Stadler M Suetrong S Takahashi H Takahashi T Tanaka K Tang LP Thambugala K Thanakitpipattana D Theodorou M Thongbai B Thummarukcharoen T Tian Q Tibpromma S Verbeken A Vizzini A Vlasák J Voigt K Wanasinghe DN Wang Y Weerakoon G Wen HA Wen TC Wijayawardene N Wongkanoun S Wrzosek M Xiao YP Xu JC Yan JY Yang J Yang SD Hu Y Zhang JF Zhao J Zhou LW Persoh D Phillips AJL Maharachchikumbura S Amoozegar MA (2016) Fungal diversity notes 253–366: Taxonomic and phylogenetic contributions to fungal taxa. Fungal Diversity 78(1): 1–237. 10.1007/s13225-016-0366-9

[B34] Lin WJ Lee YI Liu SL Lin CC Chung TY Chou JY (2020) Evaluating the tradeoffs of a generalist parasitoid fungus, *Ophiocordycepsunilateralis*, on different sympatric ant hosts. Scientific Reports 10: 6428. 10.1038/s41598-020-63400-1 PMC7156370 32286458

[B35] Luangsa-ard JJ Tasanathai K Thanakitpipattana D Khonsanit A Stadler M (2018) Novel and interesting *Ophiocordyceps* spp. (Ophiocordycipitaceae, Hypocreales) with superficial perithecia from Thailand. Studies in Mycology 89(1): 125–142. 10.1016/j.simyco.2018.02.001 29910519 PMC6002337

[B36] McNeill J Barrie FR Buck WR ARG Gantner Verlag KG (2012) International Code of Nomenclature for algae, fungi, and plants (Melbourne Code). Koeltz Scientific Books, Königstein [Regnum vegetabile no. 154].

[B37] Minh BQ Nguyen MA von Haeseler A (2013) Ultrafast approximation for phylogenetic bootstrap. Molecular Biology and Evolution 30: 1188–1195. 10.1093/molbev/mst024 23418397 PMC3670741

[B38] Mongkolsamrit S Noisripoom W Arnamnart N Lamlertthon S Himaman W Jangsantear P Samson RA Luangsa-ard JJ (2019) Resurrection of *Paraisaria* in the Ophiocordycipitaceae with three new species from Thailand. Mycological Progress 18(9): 1213–1230. 10.1007/s11557-019-01518-x

[B39] Nguyen LT Schmidt HA von Haeseler A Minh BQ (2015) IQ-TREE: A fast and effective stochastic algorithm for estimating maximum-likelihood phylogenies. Molecular Biology and Evolution 32(1): 268–274. 10.1093/molbev/msu300 25371430 PMC4271533

[B40] Patil S Sarraf G Kharkwal AC (2021) Panorama of *Metarhizium*: host interaction and its uses in biocontrol and plant growth promotion. Symbiotic Soil Microorganisms: Biology and Applications, 289–318. 10.1007/978-3-030-51916-2

[B41] Peng XC Wen TC Wei DP Liao YH Wang Y Zhang X Wang GY Zhou Y Tangtrakulwanich J Liang JD (2024) Two new species and one new combination of *Ophiocordyceps* (Hypocreales, Ophiocordycipitaceae) in Guizhou. MycoKeys 102: 245. 10.3897/mycokeys.102.113351 PMC10921062 38463694

[B42] Petch T (1931) Notes on entomogenous fungi. Transactions of the British Mycological Society 16: 55–75. 10.1016/S0007-1536(31)80006-3

[B43] Petch T (1937) Notes on entomogenous fungi. Transactions of the British Mycological Society 21: 34–67. 10.1016/S0007-1536(37)80005-4

[B44] Petch T (1939) Notes on entomogenous fungi. Transactions of the British Mycological Society 23: 127–148. 10.1016/S0007-1536(33)80026-X

[B45] Pu SC Qin L Chen MJ Cai Y Huang B (2013) Host shift and host specificity analysis of Beauveria bassiana in Masson’s pine plantation based on SSR molecular marker. Mycosystema 32(4): 698–709

[B46] Qu JJ Zou X Cao W Xu ZS Liang ZQ (2021) Two new species of *Hirsutella* (Ophiocordycipitaceae, Sordariomycetes) that are parasitic on lepidopteran insects from China. MycoKeys 82: 81–96. 10.3897/mycokeys.82.66927 34408539 PMC8367965

[B47] Qu JJ Yu LQ Zhang J Han YF Zou X (2018) A new entomopathogenic fungus, *Ophiocordycepsponerus* sp. nov., from China. Phytotaxa 343(2): 116–126. 10.11646/phytotaxa.343.2.2

[B48] Quandt CA Kepler RM Gams W Araújo JP Ban S Evans HC Hughes D Humber R Hywel-Jones N Li Z Luangsa-ard JJ Rehner SA Sanjuan T Sato H Shrestha B Sung GH Yao YJ Zare R Spatafora JW (2014) Phylogenetic-based nomenclatural proposals for Ophiocordycipitaceae (Hypocreales) with new combinations in *Tolypocladium*. IMA Fungus 5: 121–134. 10.5598/imafungus.2014.05.01.12 25083412 PMC4107890

[B49] Ronquist F Teslenko M van der Mark P Ayres DL Darling A Höhna S Larget B Liu L Suchard MA Huelsenbeck JP (2012) MrBayes 3.2: Efficient Bayesian phylogenetic inference and model choice across a large model space. Systematic Biology 61: 539–542. 10.1093/sysbio/sys029 22357727 PMC3329765

[B50] Saltamachia SJ Araújo JP (2020) *Ophiocordycepsdesmidiospora*, a basal lineage within the “Zombie-Ant Fungi” clade. Mycologia 112(6): 1171–1183. 10.1080/00275514.2020.1732147 32484758

[B51] Sanjuan TI Franco-Molano AE Kepler RM Spatafora JW Tabima J Vasco-Palacios AM Restrepo S (2015) Five new species of entomopathogenic fungi from the Amazon and evolution of neotropical *Ophiocordyceps*. Fungal Biology 119(10): 901–916. 10.1016/j.funbio.2015.06.010 26399185

[B52] Schoch CL Seifert KA Huhndorf S Robert V Spouge JL Levesque CA Chen Wen Chen w Fungal Barcoding Consortium (2012) Nuclear ribosomal internal transcribed spacer (ITS) region as a universal DNA barcode marker for Fungi. Proceedings of the National Academy of Sciences of the United States of America 109(16): 6241–6246. 10.1073/pnas.1117018109 22454494 PMC3341068

[B53] Simmons DR Kepler RM Renner SA Groden E (2015) Phylogeny of *Hirsutella* species (Ophiocordycipitaceae) from the USA: Remedying the paucity of *Hirsutella* sequence data. IMA Fungus 6(2): 345–356. 10.5598/imafungus.2015.06.02.06 26734545 PMC4681258

[B54] Spatafora JW Sung GH Sung JM Hywel‐Jones NL White Jr JF (2007) Phylogenetic evidence for an animal pathogen origin of ergot and the grass endophytes. Molecular Ecology 16(8): 1701–1711. 10.1111/j.1365-294X.2007.03225.x 17402984

[B55] Sun T Zou W Dong Q Huang O Tang D Yu H (2022) Morphology, phylogeny, mitogenomics and metagenomics reveal a new entomopathogenic fungus *Ophiocordycepsnujiangensis* (Hypocreales, Ophiocordycipitaceae) from Southwestern China. MycoKeys 94: 91. 10.3897/mycokeys.94.89425 PMC9836510 36760544

[B56] Sung GH Spatafora JW Zare R Hodge KT Gams W (2001) A revision of Verticilliumsect. Prostrata. II. Phylogenetic analyses of SSU and LSU nuclear rDNA sequences from anamorphs and teleomorphs of the Clavicipitaceae. Nova Hedwigia 72(3–4): 311–328. 10.1127/nova.hedwigia/72/2001/311

[B57] Sung GH Hywel-Jones NL Sung JM Luangsa-ard JJ Shrestha B Spatafora JW (2007) Phylogenetic classification of *Cordyceps* and the clavicipitaceous fungi. Studies in Mycology 57: 50–59. 10.3114/sim.2007.57.01 PMC2104736 18490993

[B58] Tang D Zhu J Luo L Hou D Wang Z Yang S Yu H (2022) *Ophiocordycepsovatospora* sp. nov.(Ophiocordycipitaceae, Hypocreales), pathogenic on termites from China. Phytotaxa 574(1): 105–117. 10.11646/phytotaxa.574.1.8

[B59] Tasanathai K Thanakitpipattana D Himaman W Phommavong K Dengkhhamounh N Luangsa-ard J (2020) Three new Ophiocordyceps species in the *Ophiocordycepspseudoacicularis* species complex on Lepidoptera larvae in Southeast Asia. Mycological Progress 19(10): 1043–1056. 10.1007/s11557-020-01611-6

[B60] Tian MY Huang SB (2015) Two new species of cavernicolous trechines from southern China karst (Coleoptera: Carabidae: Trechinae). Journal of Caves and Karst Studies 77(3): 152–159.

[B61] Tzean SS Hsieh LS Wu WJ (1997) The genus *Gibellula* on spiders from Taiwan. Mycologia 89: 309–318. 10.1080/00275514.1997.12026787

[B62] Wang L Li HH Chen YQ Zhang WM Qu LH (2014) *Polycephalomyceslianzhouensis* sp. nov., a new species, co-occurs with *Ophiocordycepscrinalis*. Mycological Progress 13(4): 1089–1096. 10.1007/s11557-014-0996-9

[B63] Wang YB Yu H Dai YD Wu CK Zeng WB Yuan F Liang ZQ (2015b) *Polycephalomycesagaricus*, a new hyperparasite of *Ophiocordyceps* sp. infecting melolonthid larvae in southwestern China. Mycological Progress 14: 1–9. 10.1007/s11557-015-1090-7

[B64] Wang YW Hong TW Tai YL Wang YJ Tsai SH Lien PTK Chou TH Lai JY Chu R Ding ST Irie K Li TK Tzean SS Shen TL (2015a) Evaluation of an epitypified *Ophiocordycepsformosana* (*Cordyceps**s.l.*) for its pharmacological potential. evidence-based complementary and alternative medicine 2015(1): 189891. 10.1155/2015/189891 26451152 PMC4587430

[B65] Wang YB Nguyen TT Dai YD Yu H Zeng WB Wu CK (2018) Molecular phylogeny and morphology of *Ophiocordycepsunituberculata* sp. nov. (Ophiocordycipitaceae), a pathogen of caterpillars (Noctuidae, Lepidoptera) from Yunnan, China. Mycological Progress 17: 745–753. 10.1007/s11557-017-1370-5

[B66] Wang Y Dai YD Yang ZL Guo R Wang YB Zhu LY Ding L Yu H (2021) Morphological and molecular phylogenetic data of the Chinese medicinal fungus *Cordycepsliangshanensis* reveal its new systematic position in the family Ophiocordycipitaceae. Mycobiology 9: 1–11. 10.1080/12298093.2021.1923388 PMC8409936 34512076

[B67] Wen TC Zhu RC Kang JC Huang MH Tan DB Ariyawansha H Hyde KD Liu H (2013) *Ophiocordycepsxuefengensis* sp. nov. from larvae of *Phassusnodus* (Hepialidae) in Hunan Province, southern China. Phytotaxa 123(1): 41–50. 10.11646/phytotaxa.123.1.2

[B68] Wen TC Xiao YP Li WJ Kang JC Hyde KD (2014) Systematic analyses of *Ophiocordycepsramosissimum* sp. nov., a new species from a larvae of Hepialidae in China. Phytotaxa 161(3): 227–234. 10.11646/phytotaxa.161.3.6

[B69] Wichadakul D Kobmoo N Ingsriswang S Tangphatsornruang S Chantasingh D Luangsa-ard JJ Eurwilaichitr L (2015) Insights from the genome of *Ophiocordycepspolyrhachis-furcata* to pathogenicity and host specificity in insect fungi. BMC Genomics 16: 1–14. 10.1186/s12864-015-2101-4 26511477 PMC4625970

[B70] Xiao YP Wen TC Hongsanan S Sun JZ Hyde KD (2017) Introducing *Ophiocordycepsthanathonensis*, a new species of entomogenous fungi on ants, and a reference specimen for *O.pseudolloydii*. Phytotaxa 328(2): 115–126. 10.11646/phytotaxa.328.2.2

[B71] Xiao YP Wang YB Hyde KD Eleni G Sun JZ Yang Y Meng J Yu H Wen TC (2023) Polycephalomycetaceae, a new family of clavicipitoid fungi segregates from Ophiocordycipitaceae. Fungal Diversity 120(1): 1–76. 10.1007/s13225-023-00517-4

[B72] Xu M Ashley NA Vaghefi N Wilkinson I Idnurm A (2023) Isolation of strains and their genome sequencing to analyze the mating system of *Ophiocordycepsrobertsii*. PLoS ONE 18(5): e0284978. 10.1371/journal.pone.0284978 PMC10153710 37130139

[B73] Yang ZL Qin J Xia C Hu Q Li QQ (2015) *Ophiocordycepshighlandensis*, a new entomopathogenic fungus from Yunnan, China. Phytotaxa 204(4): 287–295. 10.11646/phytotaxa.204.4.5

[B74] Zha LS Kryukov VY Ding JH Jeewon R Chomnunti P (2021) Novel taxa and species diversity of *Cordyceps**sensu lato* (Hypocreales, Ascomycota) developing on wireworms (Elateroidea and Tenebrionoidea, Coleoptera). MycoKeys 78: 79–117. 10.3897/mycokeys.78.61836 33854402 PMC8021543

[B75] Zhang S Zhang YJ Liu XZ Zhang H Liu DS (2013) On the reliability of DNA sequences of *Ophiocordycepssinensis* in public databases. Journal of Industrial Microbiology & Biotechnology 40: 365–378. 10.1007/s10295-012-1228-4 23397071

[B76] Zhang L Zhou Z Guo Q Fokkens L Miskei M Pócsi I Zhang W Chen M Wang L Sun Y Donzelli BGG Gibson DM Nelson DR Luo JG Rep M Liu H Yang SN Wang J Krasnoff SB Xu YQ Molnár I Lin M (2016) Insights into adaptations to a near-obligate nematode endoparasitic lifestyle from the finished genome of *Drechmeriaconiospora*. Scientific Reports 6(1): 23122. 10.1038/srep23122 26975455 PMC4792172

[B77] Zhang DF Gao FL Jakovlić I Zou H Zhang J Li WX Wang GT (2020) PhyloSuite: An integrated and scalable desktop platform for streamlined molecular sequence data management and evolutionary phylogenetics studies. Molecular Ecology Resources 20(1): 348–355. 10.1111/1755-0998.13096 31599058

[B78] Zhu GS Liu ZY Wu XL Liu YX (2004) The research of *Cordyceps* in the karst area of Yunnan, Guizhou and Guangxi Province in China. Guizhou Science 22(1): 27–30.

[B79] Zou X Liu AY Liang ZQ Han YF Yang MF (2010) *H.liboensis*, a new entomopathogenic species affecting Cossidae (Lepidoptera) in China. Mycotaxon 111: 39–44. 10.5248/111.39

[B80] Zou X Zhou JX Liang ZQ Han YF (2016a) *Hirsutellashennongjiaensis*, a new entomopathogenic species infecting *Earwig* (Dermaptera). Mycosystem 35: 1070–1079. 10.13346/j.mycosystema.160077

[B81] Zou X Zhou YM Liang ZQ Xu FL (2016b) A new species of the genus *Hirsutella* with helical twist neck of phialides parasitized on Tortricidae. Mycosystema 35(7): 807–813. 10.13346/j.mycosystema.150189

[B82] Zou X Qu JJ Han YF Liang ZQ (2021a) Two new species of entomopathogenic fungi with synnemata. Journal of Mountain Agriculture and Biology 40(6): 1–12.

[B83] Zou YT Mao KN Yuan Y (2021b) Landscape assemblage characteristics of plateau karst canyons: A case study of Malinghe area of Xingyi, Guizhou. Science and Technology Innovation and Application 11(36): 42–45. 10.19981/j.CN23-1581/G3.2021.36.011

