# Supplementary figures and images for: ﻿Description of two new species of Ophiocordyceps: O.sinocampes and O.cystidiata (Ophiocordycipitaceae, Hypocreales) from typical karst landform forests in Guizhou, China
Source: MycoKeys. 2025 Feb 13;114:1–27. doi: 10.3897/mycokeys.114.134323 (PMC11843171; doi:10.3897/mycokeys.114.134323)

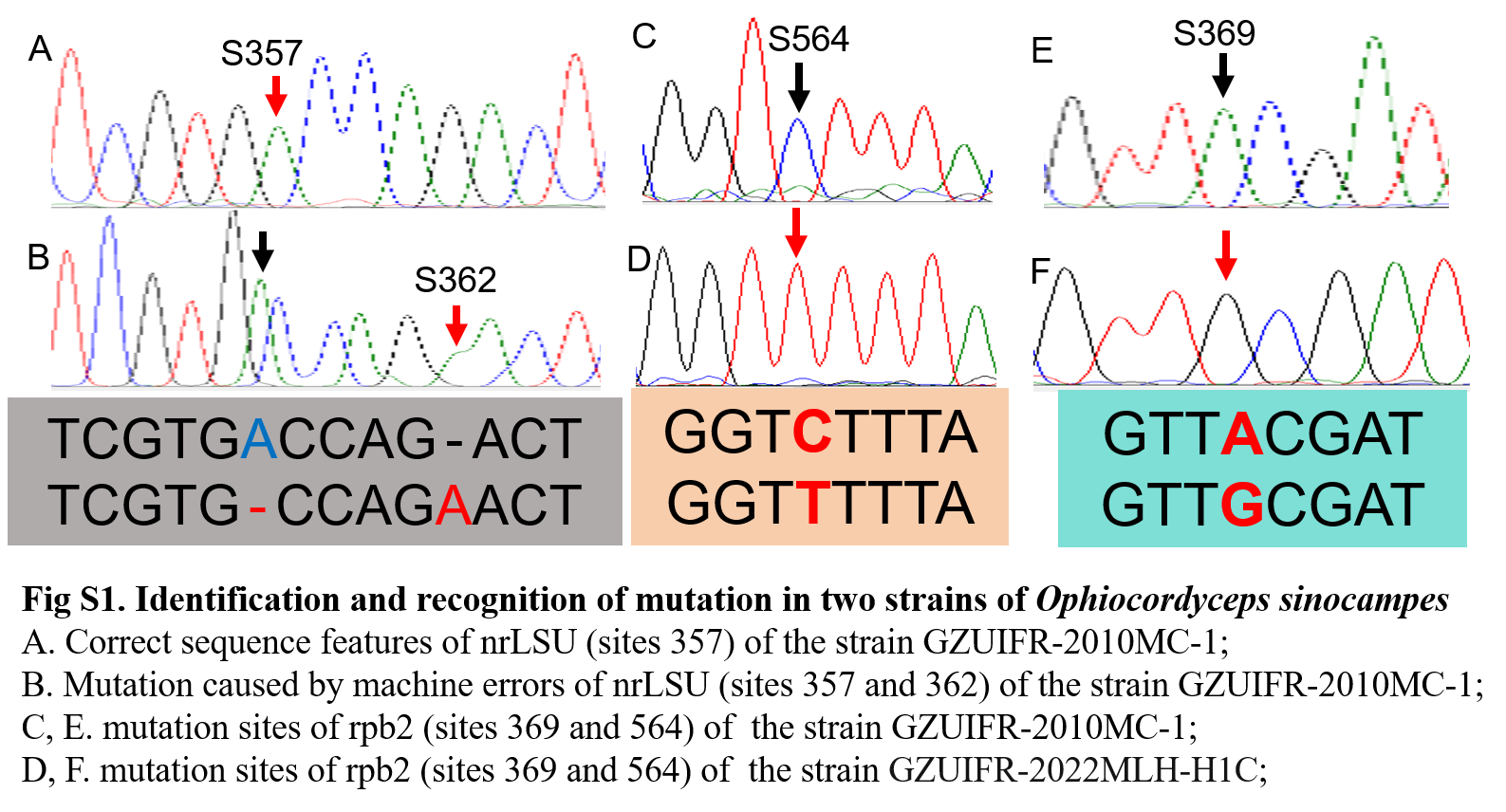

Supplement: Supplementary material 2 — Identification and recognition of mutation in two strains of Ophiocordycepssinocampes [file mycokeys-114-001-s002.png]
